# Lactic Acid Treatment of Cereals and Dietary Phytase Modified Fecal Microbiome Composition Without Affecting Expression of Virulence Factor Genes in Growing Pigs

**DOI:** 10.3389/fmicb.2019.02345

**Published:** 2019-10-15

**Authors:** Jutamat Klinsoda, Julia Vötterl, Qendrim Zebeli, Barbara U. Metzler-Zebeli

**Affiliations:** ^1^Institute of Animal Nutrition and Functional Plant Compounds, University of Veterinary Medicine, Vienna, Austria; ^2^Institute of Food Research and Product Development, Kasetsart University, Bangkok, Thailand

**Keywords:** fecal microbiome, phytase, lactic acid, virulence factor expression, pig, metabolically active bacteria

## Abstract

Besides the major nutrients, phosphorus (P) is an essential mineral for both the host animal and the porcine gut microbiota. Different strategies including phytase supplementation and more recently lactic acid (LA) are used to enhance the P availability from cereals in pig diets; however, their impact on the gut microbiota has been rarely related to fecal shedding of opportunistic pathogens. The present study investigated the effect of phytase supplementation and the treatment of dietary cereals with 2.5% LA on the fecal microbiome composition of metabolic active bacteria and expression of virulence factor genes of enterotoxigenic *Escherichia coli* and *Clostridium perfringens* in growing pigs. Phytase supplementation reduced the fecal abundance of the most abundant *Lactobacillaceae* family, whereas the LA-treatment of cereals had a stronger impact on the bacterial community, reducing amylolytic, pullulanolytic and hemicellulolytic *Lactobacillaceae*, *Lachnospiraceae* and *Ruminococcaceae* as well as the fecal bacterial species richness (Chao1) and diversity (Shannon index). Mainly the family *Clostridiaceae* benefited from the decline in the aforementioned families, being enriched by both dietary treatments. Multigroup data integration using sparse partial least squares-discriminant analysis showed that among the most discriminative operational taxonomic units (OTU) especially two unclassified *Clostridiaceae*-OTUs, one *Prevotella copri*-like OTU and one OTU within the *vadinCA11* group were associated with calcium and P levels but were negatively linked with complex carbohydrates in feces. Heat-stable toxin A (*Sta*) of enterotoxigenic *E. coli* and *Stx2e* of Shiga-toxin producing *E. coli* were expressed in feces but were similar among feeding groups. Without modifying the total bacterial gene copies and virulence factor expression of *E. coli*, both dietary phytase supplementation and LA-treatment of cereals drastically altered the bacterial community composition in pig feces. Results thereby allowed for the characterization of bacterial nutrient dependencies, indicating a link between fecal P availability, complex carbohydrate composition and alterations in the predominant genera.

## Introduction

Aside from the major nutrients, the dietary supply of minerals, such as calcium (Ca) and phosphorus (P), modifies the porcine intestinal microbiota (Mann et al., 2014; Heyer et al., 2015). Both Ca and P are essential nutrients for the bacterial cell metabolism, for instance, in being constituents of nucleotides, co-factors, teichoic acids and phospholipids (Metzler and Mosenthin, 2008). We could previously show that different intestinal P availabilities altered the cellulolytic activity (Metzler et al., 2008) and composition of the bacterial microbiota along the gastrointestinal tract of pigs (Metzler-Zebeli et al., 2010, 2013; Mann et al., 2014). Due to environmental concerns associated with high P levels in manure, the total P content of pig diets has been decreased over the past two decades (Oster et al., 2017). This has not only reduced the total P available for the pig but also for the intestinal microbiota. Most P in plant feedstuffs is in the form of phytate-P (Cowieson et al., 2017). Albeit intestinal bacteria can utilize phytate-P, they only increase their phytase activity when the available P in their surroundings becomes low (Dersjant-Li et al., 2015).

As phytate-P cannot be hydrolyzed by mammalian enzymes (Cowieson et al., 2017), different strategies are applied to enhance the dietary P availability and consequently prevent P deficiencies in pigs, mainly dietary supplementation of phytase (Metzler-Zebeli et al., 2010) or (less frequently used) soaking and fermentation of cereals (Blaabjerg et al., 2011, 2012). However, the efficacy of exogenous phytase addition may be lower due to animal (e.g., gastrointestinal pH, digesta passage rate), and feed-related factors (e.g., dietary buffering capacity and pelleting temperature) (Blaabjerg et al., 2011, 2012; Kebreab et al., 2012; Yáñez et al., 2013). For this reason, feed-technological approaches (e.g., soaking and fermentation) that enhance the phytate-P availability in cereal grains prior to feeding to the animals have received more attention lately. For instance, we could recently show that soaking barley grains in lactic acid (LA) reduced the phytate-P content in barley and altered the ruminal microbiota (Metzler-Zebeli et al., 2014, 2015). Aside from this, soaking of cereals in LA may lead to structural changes in other components, such as the starch and hemicellulose fractions (Deckardt et al., 2015), which are key substrates that may alter the fibrolytic bacterial community. Differences in the effects of soaking and fermentation processes on the intestinal microbiota and shedding of virulence factors in feces may be expected, partly due to the lag phase until bacterial fermentation and LA production sets in. In general, LA has bacteriostatic effects when added as feed additive to pig diets, reducing the intestinal abundance of opportunistic pathogens such as enterotoxigenic *Escherichia coli* and *Clostridium perfringens* (Suiryanrayna and Ramana, 2015; Koopmans et al., 2016). These pathogens, especially *E. coli* pathotypes (e.g., Shiga toxin-producing and enterotoxigenic *E. coli*), *C. perfringens*, and *Campylobacter coli*, are responsible for major economic losses in the pig industry (Toledo et al., 2012; Wells et al., 2015; Bardasi et al., 2017) and are a potent source of microbial contaminants in pork products, therefore being a risk factor for foodborne zoonotic diseases (Lamendella et al., 2011). Consequently, dietary strategies should be evaluated whether they increase or decrease intestinal numbers of opportunistic pathogens in order to formulate diets which promote intestinal health and reduce the risk of spreading zoonotic diseases.

Most studies investigating dietary effects on intestinal microbial communities were performed by targeting DNA (Lu et al., 2015). However, the extracted DNA may originate from dead or dormant bacteria and may therefore not represent the metabolically active bacteria, whereas bacterial RNA correlates better to microbial growth and metabolic activity (Westermann et al., 2012; Zhu et al., 2017).

Since hemicellulolytic and proteolytic bacteria previously increased with higher intestinal P availability in pigs (Metzler-Zebeli et al., 2010, 2013), we hypothesized that an increased phytate-P availability in the upper digestive tract due to dietary phytase supplementation or soaking of cereals grains in LA may modify the fibrolytic bacterial community in the distal large intestine and reduce the fecal numbers of common opportunistic pathogens. Due to the capability of LA to modulate the complex carbohydrate fractions in the cereals (Metzler-Zebeli et al., 2014), we further hypothesized that these changes additionally will alter the starch- and fiber-degrading bacterial community. Therefore, this study evaluated the effect of phytase supplementation and the treatment of dietary cereals with 2.5% LA on the fecal composition of metabolic active bacteria and expression of virulence factor genes of enterotoxigenic *E. coli* and *C. perfringens* in growing pigs.

## Materials and Methods

### Animals and Experimental Design

Thirty-two castrated male pigs (Large White, 13.1 ± 2.3 kg) with an average age of 6–8 weeks were used in this study. Pigs were obtained from the University research pig farm (University of Veterinary Medicine Vienna) where they were reared from the suckling to post-weaning phase in a similar manner across the four replicate batches. Piglets were weaned at 28 days of age. Prior to the start of the experiment, all pigs received the same feeding regimen (suckling to post-weaning phase). Two days prior to the start of the experiment, pigs were moved to the experimental room and were housed into individual metabolism pens (1.0 × 1.2 m) for the whole experimental period. After this environmental adaptation, pigs were randomly assigned to one of four dietary treatments in a 2 (two phytase levels: 0 and 500 FTU/kg) × 2 (two cereal types: treated versus non-treated) factorial design. In total, there were four replicate batches with eight pigs per replicate batch. Two pigs per replicate batch received the same experimental diet, resulting in eight observations per dietary treatment at the completion of the experiment (Supplementary Figure S1). Each replicate batch consisted of 19 days with the collection of samples from the rectum occurring on days 18 and 19. Pigs had free access to demineralized water throughout the experiment. Pigs were housed in an environmentally controlled room, and room temperature was checked three-times daily to ensure optimal temperature for the pigs. Pigs were weighed at the beginning and end of the experimental period. The health status of the animals was monitored daily.

### Diets

The diets consisted of wheat, corn and soybean meal (Table 1) and were formulated to meet or exceed the current recommendation for nutrient requirements (Gesellschaft fur Ernahrungsphysiologie [GfE], 2006, National Research Council [NRC], 2012). Pigs were fed one of four following experimental diets: (1) control (Con) diet (Con diet) (2) control diet with phytase (Con-Phy diet) (3) diet containing LA-treated cereals (LA diet) (4) diet with phytase and LA-treated cereals (LA-Phy diet). Two mineral-vitamin premixes were formulated. According to the standard inclusion level of phytase (Dersjant-Li et al., 2015), one premix comprised microbial phytase (500 FTU/kg), whereas the other premix was without microbial phytase. For the diets with LA treatment of the cereal grains, wheat and corn were soaked in a 2.5% LA solution for 48 h, as previously published (Vötterl et al., 2019). The concentration of the LA solution of 2.5% and the incubation time of 48 h of incubation have been selected based on the results of our preceding *in vitro* study (Vötterl et al., 2019). After the 48 h of incubation, cereals were dried at 70°C for 1 h and afterward at 60°C for 23 h and ground to pass a 5-mm sieve.

**TABLE 1:** Dietary ingredients and chemical composition of experimental diets.

	**No phytase**		**Phytase**	
**Treatment of cereal grains**	**Con**	**LA**	**Con**	**LA**
**Ingredient (%)**				
Wheat	36.2	36.2	36.2	36.2
Corn	36.0	36.0	36.0	36.0
Soybean HP dehulled	22.0	22.0	22.0	22.0
Sunflower Oil	2.0	2.0	2.0	2.0
Vitamin-Mineral-Premix^*a*^	2.3	2.3	2.3	2.3
Limestone	1.1	1.1	1.1	1.1
Monocalcium phosphate	0.4	0.4	0.4	0.4
Phytase (FTU/kg)^*b*^	0	0	500	500
**Analyzed chemical composition (dry matter basis) (%)**				
Dry matter	90.0	94.2	90.0	94.2
Crude ash	5.4	4.9	5.4	4.9
Crude protein	21.3	20.2	21.6	20.5
aNDF_OM_	13.1	12.2	12.9	11.9
ADF_OM_	5.0	5.1	5.2	5.1
Resistant starch	0.8	0.8	0.9	0.8
Non-resistant starch	51.0	49.2	50.0	48.5
Phosphorus	0.5	0.5	0.5	0.5
Calcium	0.6	0.6	0.6	0.6
Calcium:phosphorus	1.2	1.2	1.2	1.2

*Con, control diet; LA, diet containing lactic acid-treated cereals. ^*a*^The Vitamin-Mineral-Premix provided per kilogram of experimental diet (Garant GmbH, Pöchlarn, Austria): vitamin A, 17.250 IE; vitamin D3, 2.299 IE; vitamin E, 160.999 mg; vitamin K3, 4.600 mg; vitamin B1, 2.299 mg; vitamin B2, 6.900 mg; vitamin B6, 3.450 mg; vitamin B12, 0.034 mg; Vitamin B3, 34.500 mg; Pantothenic acid, 17.150 mg; folic acid, 1.150 mg; biotin, 0.172 mg; Choline Chloride, 579.600 mg; Fe, 103.500 mg; Cu, 17.250 mg; Zn, 103.500 mg; Mn, 45.999 mg; J, 1.725 mg; Se, 0.517 mg. ^*b*^FTU; phytase units.*

Pigs were fed three times daily at 8:00, 12:00, and 16:00 h. Feed allowances were calculated to correspond to 3-times maintenance requirement ([(body weight^0.6^ × 197)/238.68] × 3) (Gesellschaft fur Ernahrungsphysiologie [GfE], 2006). At feeding, the experimental diets were mixed with water in a ratio of 3: 1 and immediately offered to the pigs. Feed leftovers (feed spillage and feed remainings in the feeding bowls) were collected, dried, and weighed to determine dry matter intake. Diet samples were collected in each replicate run.

### Slaughtering and Fecal Sample Collection

On days 18 and 19, pigs were euthanized 2 h after their last feeding via intracardiac injection of T61 (10 ml/kg Embutramide, MSD animal Health, Vienna, Austria) after general anesthesia was induced (Narketan 100 mg/ml, 1 ml/10 kg body weight, Ketaminhydrochlorid, Vétoquinol GmbH, Germany; Stresnil 40 mg/ml; 0.5 ml/10 kg body weight, Azaperon, Elanco Deutschland GmbH, Germany). For the analysis of metabolic active bacteria, fresh feces from the rectum were aseptically collected in 2-ml cryo-tubes (Sarstedt AG & Co., Nümbrecht, Germany), immediately snap-frozen in liquid nitrogen and stored at −80°C until analysis. In one pig, the rectum was empty and digesta could not be collected for microbial analysis.

### Chemical Analyses

Diets, freeze-dried digesta and fecal samples were analyzed for dry matter, protein, ash, calcium (Ca) and P according to VDLUFA as previously published (Metzler-Zebeli et al., 2013). The content of neutral detergent fiber (aNDF_OM_) and acid detergent fiber (ADF_OM_) were determined using Fiber Therm FT 12 (Gerhardt GmbH & Co., KG, Königswinter, Germany) (Metzler-Zebeli et al., 2014). Resistant (RS) and non-resistant (NRS) Starch contents were analyzed photometrically using enzymatic assay kits (K-RSTAR: Megazyme International Ireland, Ltd., Braz Ireland) according to the manufacturer’s instructions.

### Total RNA Extraction

Total RNA was extracted using the RNeasy PowerMicrobiome Kit following the manufacturer’s instructions (Qiagen, Hilden, Germany) with some modifications with respect to the bead beating procedure. Frozen feces (250 mg) were weighed into 2-mL screw-cap tubes, containing 0.6 g sterile glass beads (Ø 0.1 mm), 0.4 g ceramic beads (Ø 1.4 mm), 0.55 g ceramic beads (Ø 2.8 mm) and 650 μL guanidinium thiocyanate buffer. TheFastPrep-24 instrument (MP Biomedicals, Heidelberg, Germany) was used for homogenization which consisted of three bead beating steps- each one 1 min at 6.5 m/swith cooling on ice between the bead beating steps. Thereafter, samples were processed according to the manufacturer’s instructions (Qiagen). To remove genomic DNA, the extracted RNA was treated with DNase I (Turbo DNA kit, Life Technologies Limited, Vienna Austria) following the manufacturer’s instructions. The elution volume was 50 μL. The total RNA of each sample was quantified by the Qubit 2.0 Fluorometer (Life Technologies Corporation, CA, United States) using Qubit RNA Assay Kit according to the manufacturer’s instructions as well as qualified with Agilent Bioanalyzer 2100 (Agilent Technologies, Waldbronn, Germany). The Agilent RNA 6000 Nano Assay (Agilent Technologies, Waghäusel-Wiesental, Germany) was used to determine the RNA integrity numbers (RIN), which ranged between 6 and 10. The cDNA synthesis was performed using the High Capacity Reverse Transcription Kit (Life Technologies Foster City, United States) following the manufacturer’s instructions. One μg of total RNA and 0.5 μL of RNase Inhibitor (Qiagen) were added to each reaction. One aliquot of the cDNA was sent for 16S rRNA gene amplicon sequencing, whereas a second aliquot was used for absolute quantification of total bacteria and virulence factors.

### Quantitative PCR

Absolute quantification of total bacteria and virulence factor genes (*cpa*, *STa* and *STx2e*) in fecal samples was performed on a Stratagene Mx3000P qPCR system (Agilent Technologies) using previously published primer sets (Supplementary Table S1) (Metzler-Zebeli et al., 2010; Muyzer et al., 1993; Schlegel et al., 2012). Primer sequences were verified with PrimerBLAST^1^ and tested for efficiencies and specificity using melting curve analysis (Supplementary Table S1). Each 20 μl reaction consisted of 5 ng cDNA, 10 μl Eva Green master mix with low ROX (Biotium, Hayward, CA, United States), 400 nM each of forward and reverse primers, and 10 μl DEPC-treated water (Bioscience) in a 96-well plate in duplicate. The amplification protocol comprised an initial denaturation at 95°C for 5 min, followed by 40 cycles of 95°C for 15 s, primer annealing at 60°C for 30 s, and elongation at 72°C for 30 s, followed by the generation of melting curves with increments of 0.1°C/s between 55 and 95°C. Additionally, negative controls and the reverse transcription controls (RT minus) were also run on each plate.

Standard curves were prepared from 10-fold serial dilutions (10^7^ to 10^3^ molecules/μl) of the purified and quantified PCR products using genomic DNA from pig feces of the present study (Metzler-Zebeli et al., 2016). The final copy number of total bacteria and virulence factor genes was calculated using the following equation: (QM × C × DV)/(S × V), where QM is the quantitative mean of the copy number, C is the DNA concentration of each sample, DV is the dilution volume of isolated DNA, S is the DNA amount (ng) and V is the weight of the sample (g) subjected to DNA extraction. Amplification efficiencies (*E* = 10^(–1/slope)^) and coefficient of determination (linearity) can be found in Supplementary Table S1.

### 16S rRNA Sequencing and Bioinformatic Analysis

The 16S rRNA PCRs, library preparation, and sequencing using the Illumina MiSeq sequencing platform was performed by a commercial provider (Microsynth AG, Balgach, Switzerland) as previously described (Metzler-Zebeli et al., 2018). The V3-V4 hypervariable regions of bacterial 16S rRNA were amplified using the primers 341F-ill (5′-CCTACGGGNGGCWGCAG-3′) and 802R-ill (5′-GACTACHVGGGTATCTAATCC-3′) which produces an amplicon of approximately 460 bp. The 16S rRNA gene PCRs were performed using the KAPA HiFi HotStart PCR Kit (Roche, Baden, Switzerland). The Nextera XT sample preparation kit (Illumina Inc.) was used to construct libraries by ligating sequencing adapters and indices onto purified PCR products. Afterward, equimolar quantities of each library were pooled and sequenced on an Illumina MiSeq sequencing v2 platform using a 250 bp read length paired-end protocol. After sequencing, the overlapping paired-end reads were demultiplexed, trimmed of Illumina adaptor residuals using cutadapt^2^ and stitched using USEARCH (drive5/com) by Microsynth.

The stitched reads were processed using the software package Quantitative Insights into Microbial Ecology (QIIME, v1.9.4) (Caporaso et al., 2010). Fastq files were quality checked using the ‘split_libraries_fastq.py’ command for non-multiplexed Illumina fastq data with the phred score offset of 33. Chimeric sequences were identified with the UCHIME method and using USEARCH8.1 and gold. fa database, which were subsequently filtered out by the ‘filter_fasta.py’ command (Edgar, 2010; Edgar et al., 2011). Open-reference OTU picking was done at 97% similarity level using UCLUST (Edgar, 2010). Taxonomy was assigned to the OTUs using the Ribosomal Database Project (RDP) naïve Bayesian rRNA classifier and the Greengenes database as reference template (version 13.8) (De Santis et al., 2006). Rare OTUs with less than 10 sequences were excluded. In addition, the sequence identity of OTUs at >0.01% relative abundance was checked using Microbial Nucleotide BLAST^3^ (Supplementary Table S2). For α-diversity (Shannon, Simpson, and Chao1) measurements, samples were rarefied to a depth of 24,500 sequences. For β-diversity analysis, statistical assessment of dissimilarity matrices (Bray-Curtis) derived from OTU data was performed using the ‘adonis2’ function (PERMANOVA) in the R package ‘vegan’ (version 2.5.2). The PERMANOVA was applied on the Bray-Curtis distance matrices between dietary factors (phytase supplementation, LA treatment of cereals and their interaction) and statistical significance was calculated after 999 random permutations. Clustering of fecal samples from the four dietary treatment groups were visualized in two-dimensional non-metric multidimensional scaling (NMDS) ordination plots obtained with the ‘metaMDS’ function in the vegan R package (Oksanen et al., 2018) in R studio (version 1.0.136). To identify the most discriminant OTUs and nutrients in feces, multigroup supervised DIABLO N-integration networking was performed using the R package ‘mixOmics’ (version 6.3.2) (Rohart et al., 2017). Horizontal sparse partial least squares-discriminant analysis (sPLS-DA) using the ‘block.splsda’ function was applied to integrate the datasets of relative abundances of OTUs and nutrient composition in feces to classify and select key features from each dataset. This enabled the discrimination of treatment groups with the lowest possible error rate, selecting 20 OTUs which were associated with the fecal content of protein, ash, Ca and P forming the “nutrient” dataset and the fecal content of aNDF_OM_, ADF_OM_, RS and NRS forming the “carb” dataset. Results were visualized in loading plots and heatmaps for component 1 and 2 as well as in circos plots for component 1 and 2, illustrating the strongest positive and negative correlations among the “OTU”, “nutrients” and “carb” datasets.

### Statistical Analysis

Prior to the start of the experiment, a power test analysis estimated according to Kononoff and Hanford (2006) and based on previous data for the intestinal microbiota composition and interaction with the host animal (Heyer et al., 2015; Kelly et al., 2017; Metzler-Zebeli et al., 2013, 2018, 2019; Mann et al., 2014) using the SAS software (version 9.4; SAS Inst. Inc., Cary, NC, United States) was performed to identify the minimum number of observations (*n* = 6) required for the present pig experiment to reject the null-hypothesis if this was false.

To test for differences in relative abundance of bacterial taxa, only taxa appearing in at least 50% of the fecal samples were considered. The raw read counts from the tables of OTU abundances were collapsed and compositionally normalized such that each sample sums to 1. The relative abundances at the respective taxonomic rank were analyzed. All variables were tested for normal distribution by the Shapiro-Wilk test with the UNIVARIATE procedure in SAS (Version 9.4, SAS Inst. Inc., Cary, NC). To compare differences between dietary treatments, data for α-diversity, bacterial phyla, families and genera as well as selected OTUs were subjected to ANOVA using the MIXED procedure in SAS. The model included the fixed effects of phytase supplementation, LA treatment of grains and their two-way-interaction and replicate as random effect. Pig was the experimental unit. The degrees of freedom were approximated by the Kenward-Rogers method (ddfm = kr). The means were reported as least-squares means ± standard error of the mean (SEM). The differences were considered significant if *p* < 0.05 and trends at 0.05 < *p* ≤ 0.10. However, the discussion was mostly based on significant changes between dietary treatments.

## Results

### Pigs, Dietary and Fecal Chemical Composition

All pigs remained healthy throughout the study and ate the same amount of feed across treatment groups (784 ± 20.4 g/day). The LA treatment of cereals reduced the ash, protein, and aNDF_OM_ contents of the diets by 0.5, 1.1 and 1.0%, respectively (Table 1). The LA treatment increased the dry matter (DM) content of feces (*p* < 0.001), whereas the phytase supplementation tended to reduce it (Table 2; *p* < 0.10). Treating cereals with LA also increased (*p* = 0.004) the aNDF_OM_ content in pig feces by 1.9%. Both phytase supplementation and LA treatment of the cereal grains increased the ADF_OM_ content in feces (*p* < 0.05). The concentration of total, RS and NRS fractions tended to be higher in feces of pigs fed the LA-treated cereals (*p* < 0.10). When expressing the RS and NRS fractions as proportion of total starch, the RS fraction was smaller with the LA-treated cereals. Moreover, both phytase supplementation and LA treatment decreased the fecal P content, whereas only the phytase supplementation lowered the Ca content and subsequently enhanced the Ca:P ratio in feces (*p* < 0.001).

**TABLE 2:** Characteristic of feces from pigs fed diets with or without phytase and lactic acid treatment of cereals.

	**No phytase**		**Phytase**			***p*-value**^1^		
**Treatment of cereal grains**	**Con**	**LA**	**Con**	**LA**	**SEM**	**Phytase**	**LA**	**Phytase × LA**
Dry matter (%)	30.6	34.9	28.1	31.2	1.666	0.076	0.038	0.696
Crude Protein (%)	23.1	23.9	23.0	23.0	0.430	0.271	0.400	0.319
Neutral-detergent fiber (%)	39.7	41.5	40.8	42.7	0.591	0.058	0.004	0.944
Acid-detergent fiber (%)	16.7	17.4	17.5	18.0	0.242	0.010	0.022	0.605
Total starch (%)	5.64	7.00	6.69	7.88	0.737	0.202	0.096	0.913
Non-resistant starch (%)	5.5	6.8	6.5	7.7	0.738	0.205	0.091	0.915
Resistant starch (%)	0.17	0.15	0.18	0.15	0.010	0.607	0.068	0.811
Phosphorus (g/kg)	2.1	1.9	1.3	1.1	0.406	< 0.001	<0.001	0.891
Calcium (g/kg)	1.7	1.5	1.1	1.1	0.539	< 0.001	0.242	0.205
Calcium: Phosphorus-ratio	0.78	0.79	0.84	1.01	0.026	< 0.001	<0.001	0.005

*Values are presented as least square means ± SEM (*n* = 8 pigs per dietary treatment). Con, control diet; LA, diet containing lactic acid-treated cereals. ^1^*p*-values for fixed effects (phytase, LA treatment of cereals and their two-way interaction) were computed using ANOVA and the PROC MIXED in SAS.*

### Bacterial Community

The absolute abundance of metabolically active bacteria in feces amounted to 11.1 log_10_ gene copies/g feces (Table 3). After quality and chimera filtering, a total of 1,569,539 reads with a mean of 49,048 ± 17,836 sequences per sample and a mean read length of 403.39 ± 35.60 bp remained. Reads were clustered into 3,545 operational taxonomical units (OTUs).

**TABLE 3:** Absolute abundance of total bacterial 16S rRNA and virulence factors (log_10_ gene copies/g) in feces of pigs fed diets with or without phytase and lactic acid treatment of cereals.

	**No phytase**		**Phytase**			***p*-value**^1^		
**Treatment of cereal grains**	**Con**	**LA**	**Con**	**LA**	**SEM**	**Phytase**	**LA**	**Phytase × LA**
Total bacteria	11.3	11.0	11.1	11.2	0.29	0.926	0.625	0.544
ETEC *STa*	5.03	4.86	5.03	4.99	0.18	0.705	0.576	0.708
STEC *Stx2e*	4.7	4.6	4.8	4.7	0.19	0.627	0.613	0.742

*Values are presented as least square means ± SEM (*n* = 8 pigs per dietary treatment; with exception of Con diet with phytase group, *n* = 7). ETEC, enterotoxigenic *Escherichia coli*, STa, heat-stable enterotoxin, STEC, Shiga toxin *E. coli*, Stx2e, Shiga toxin type 2e. Con, control diet; LA, diet containing lactic acid-treated cereals. ^1^*p*-values for fixed effects (phytase, LA treatment of cereals and their two-way interaction) were computed using ANOVA and the PROC MIXED in SAS.*

The most abundant phyla in feces were *Firmicutes* (78.8% of all reads), followed by *Euryarchaeota* (13.0% of all reads) and *Bacteroidetes* (4.2% of all reads) (Supplementary Table S3). Phylogenetic classification identified 12 families with a mean abundance greater than 0.01% of all reads (Table 4), with *Lactobacillaceae* (20.1% of all reads), *Clostridiaceae* (19.3% of all reads), *Lachnospiraceae* (16.7% of all reads), and *Ruminococcaceae* (13.0% of all reads) being the dominant families. Accordingly, *Lactobacillus* and unclassified genera within *Clostridiaceae*, *Lachnospiraceae*, and *Ruminococcaceae* were the dominant genera (Supplementary Table S4). The same trends were also found at operational taxonomic unit (OTU) level with a relative abundance of >0.01% of all reads (Supplementary Table S5).

**TABLE 4:** Relative abundance (%) of bacterial families (>0.01% of all reads) in feces of pigs fed diets with or without phytase and lactic acid treatment of cereals.

	**No phytase**		**Phytase**			***p*-value**^1^		
**Treatment of cereal grains**	**Con**	**LA**	**Con**	**LA**	**SEM**	**Phytase**	**LA**	**Phytase × LA**
*Lactobacillaceae*	25.88	25.91	16.23	12.43	4.703	0.022	0.693	0.688
*Clostridiaceae*	9.22	16.85	18.02	33.13	5.393	0.029	0.046	0.495
*Lachnospiraceae*	18.09	13.42	22.85	12.59	2.858	0.498	0.015	0.338
*Ruminococcaceae*	16.89	12.49	14.01	8.78	1.788	0.078	0.013	0.818
*Methanobacteriaceae*	12.82	13.91	12.40	11.61	2.447	0.582	0.951	0.704
*Prevotellaceae*	2.34	3.03	1.66	6.97	1.806	0.376	0.110	0.214
Unclassified *Clostridiales*-1	3.07	2.11	3.68	2.28	0.513	0.446	0.031	0.672
*Veillonellaceae*	1.93	1.98	1.60	2.12	0.583	0.866	0.629	0.691
*Coriobacteriaceae*	1.38	2.85	0.64	1.99	0.599	0.194	0.027	0.923
*Christensenellaceae*	0.99	1.51	1.35	0.67	0.345	0.489	0.829	0.096
*Streptococcaceae*	1.97	0.035	2.35	0.015	1.143	0.874	0.074	0.860
*Spirochaetaceae*	0.44	1.02	0.49	1.80	0.538	0.452	0.093	0.501
*Erysipelotrichaceae*	0.43	0.77	0.10	1.15	0.321	0.936	0.041	0.274
*Peptostreptococcaceae*	0.72	0.20	0.76	0.36	0.116	0.397	0.001	0.583
Unclassified *Clostridiales-*2	0.35	0.45	0.61	0.46	0.159	0.417	0.857	0.442
*Mogibacteriaceae*	0.43	0.41	0.50	0.41	0.060	0.562	0.352	0.493
*Desulfovibrionaceae*	0.36	0.23	0.40	0.30	0.091	0.563	0.224	0.869
*Methanomassiliicoccaceae*	0.38	0.14	0.50	0.25	0.099	0.256	0.021	0.970
*f__S24-7*	0.25	0.45	0.15	0.33	0.155	0.494	0.233	0.969
*Turicibacteraceae*	0.068	0.092	0.25	0.75	0.214	0.061	0.234	0.280
*Peptococcaceae*	0.27	0.33	0.20	0.12	0.056	0.021	0.831	0.241
*Paraprevotellaceae*	0.22	0.21	0.16	0.26	0.094	0.948	0.680	0.566
Unclassified *GMD14H09*	0.19	0.16	0.30	0.15	0.100	0.625	0.362	0.576
Unclassified *Bacteroidales*-1	0.16	0.21	0.20	0.22	0.078	0.744	0.643	0.792
*Succinivibrionaceae*	0.18	0.30	0.075	0.053	0.122	0.168	0.700	0.574
*Bifidobacteriaceae*	0.22	0.15	0.035	0.016	0.113	0.177	0.674	0.802
*Campylobacteraceae*	0.14	0.12	0.10	0.061	0.034	0.161	0.335	0.794
*Dehalobacteriaceae*	0.061^bc^	0.090^ab^	0.094^a^	0.055^c^	0.012	0.920	0.700	0.009
*Enterobacteriaceae*	0.111	0.131	0.027	0.013	0.050	0.057	0.949	0.741
*Eubacteriaceae*	0.031	0.027	0.000	0.197	0.066	0.302	0.156	0.139
*Porphyromonadaceae*	0.035	0.011	0.016	0.034	0.012	0.871	0.833	0.098
*Pirellulaceae*	0.017	0.019	0.030	0.026	0.013	0.438	0.923	0.769
Unclassified *YS2*	0.016	0.013	0.012	0.035	0.008	0.252	0.217	0.095
*Pasteurellaceae*	0.020	0.015	0.016	0.021	0.008	0.922	0.965	0.513
*Dethiosulfovibrionaceae*	0.016	0.016	0.014	0.005	0.007	0.358	0.534	0.542
*Sphaerochaetaceae*	0.011^ab^	0.006^ab^	0.006^b^	0.014^a^	0.003	0.635	0.546	0.026
*Helicobacteraceae*	0.009	0.009	0.007	0.008	0.002	0.565	0.870	0.836

*Values are presented as least square means ± SEM (*n* = 8 pigs per dietary treatment; with exception of Con diet with phytase group, *n* = 7 pigs). Con, control diet; LA, diet containing lactic acid-treated cereals. ^*a,b,c*^Different superscripts within a row indicate significant difference (*p* ≤ 0.05). ^1^*p*-values for fixed effects (phytase, LA treatment of cereals and their two-way interaction) were computed using ANOVA and the PROC MIXED in SAS.*

### Diet-Related Changes in Fecal Community

**FIGURE 1:**
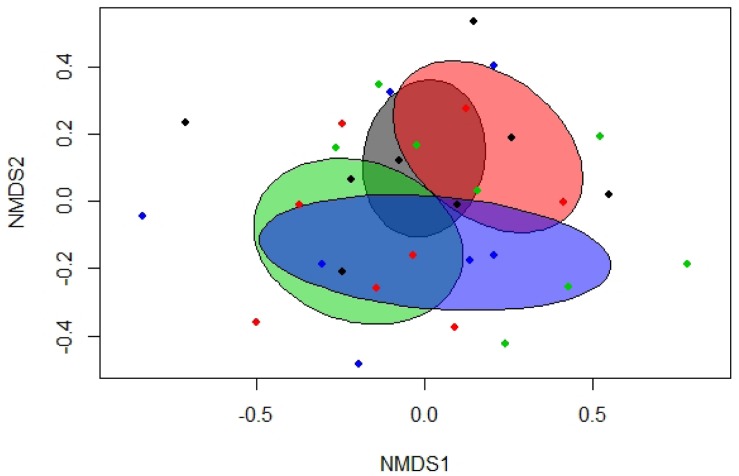
Non-metric multidimensional scaling (NMDS) plot of pairwise Bray-Curtis dissimilarities between bacterial communities in feces of pigs fed diets with or without phytase and lactic acid treatment of cereals (stress level = 0.19). Ellipses represent the standard deviation. Gray, control diet; green, diet containing lactic acid-treated cereals; red, diet with phytase supplementation; and blue, diet with phytase supplementation and lactic acid-treated cereals.

Total bacterial gene copy numbers were similar among treatment groups (Table 3). Separation between treatment groups was detected in the Bray-Curtis derived dissimilarity matrix for the bacterial communities. Accordingly, the communities of pigs fed the diets with the LA-treated cereal grains clustered apart from those of pigs fed diets with non-treated cereal grains (*p* = 0.031; *R*^2^ = 0.064). Similarly, the bacterial communities of pigs fed the diets with and without phytase, respectively, tended to cluster separately (Figure 1 and Supplementary Table S6; *p* < 0.10). This was also reflected by the α-diversity, showing a lower species richness (Chao 1) and diversity (Shannon) in feces of pigs fed the diets with the LA-treated cereals compared to pigs fed the diets with the non-treated cereals (Table 5; *p* < 0.05).

**TABLE 5:** The alpha-diversity indices in feces of pigs fed diets with or without phytase and lactic acid treatment of cereals.

	**No phytase**		**Phytase**			***p*-value**^1^		
**Treatment of cereal grains**	**Con**	**LA**	**Con**	**LA**	**SEM**	**Phytase**	**LA**	**Phytase × LA**
Shannon	6.25	5.79	6.49	5.85	0.257	0.543	0.043	0.727
Simpson	0.92	0.92	0.96	0.93	0.016	0.192	0.306	0.496
Chao1	1719	1506	1760	1470	100.8	0.983	0.020	0.706

*Values are presented as least square means ± SEM (*n* = 8 pigs per dietary treatment; with exception of Con diet with phytase group, *n* = 7 pigs). Con, control diet; LA, diet containing lactic acid-treated cereals. ^1^*p*-values for fixed effects (phytase, LA treatment of cereals and their two-way interaction) were computed using ANOVA and the PROC MIXED in SAS.*

Although no differences were detected at phylum level (Supplementary Table S3), the phytase supplementation decreased the relative abundance of *Lactobacillaceae* and *Peptococcaceae* by 0.4 and 0.3-fold, whereas it increased the abundance of *Clostridiaceae* by 1-fold (Table 4; *p* < 0.05). This was reflected at genera level where the phytase supplementation lowered the abundance of the genus *Lactobacillus* and increased the abundance of *Clostridium* (*P* < 0.05). The LA treatment of grains, in turn, only increased the abundance of *Actinobacteria* by 0.9-fold at phylum level (*p* = 0.04), whereas alterations in the relative abundances of bacterial taxa could be found within the *Firmicutes* without changing the abundance of the phylum as a whole. Overall, the LA treatment of cereals affected 8 bacterial families (>0.01% of all reads) and 13 genera (>0.05% of all reads), with 3 families and 4 genera being increased and 5 families and 9 genera being decreased, respectively (Table 4 and Supplementary Table S4). Drastic changes were observed within the predominant *Firmicutes* families, with *Clostridiaceae* increasing, and the other families decreasing in feces of pigs fed the LA diets (*p* < 0.05).

### Expression of Virulence Factor Genes in Feces

In using the compositional information of the metabolically active bacteria from the sequencing data, we quantified the abundances of genes for the heat-stable toxin A (*STa*) and Shiga toxin STEC (*Stx2e*) of *E. coli* as well as for α-toxin (*cpa*) of *C. perfringens* in order to investigate the impact of phytase supplementation and LA treatment of cereal grains on their fecal expression. In feces, expression of *STa* (4.9 log_10_ gene copies/g) and *Stx2e* (4.7 log_10_ gene copies/g) was detected (Table 3), whereas *cpa* cDNA was below detection limit, indicating that no or only few gene transcripts were present in feces. The dietary treatments had no effect on the gene copy numbers of *STa* and *Stx2e*.

### Associations Between OTUs and Nutrient Availability in Feces

**FIGURE 2:**
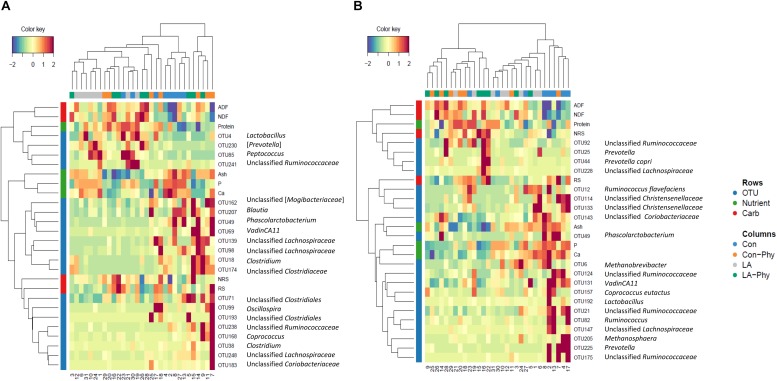
Clustered Image Maps (Euclidean distance, complete linkage) of the multi-omics signature for the identified best discriminant operational taxonomic units (OTUs; *n* = 20; relative OTU abundance >0.04%), complex carbohydrates (RS, NRS, aNDF_OM_ and ADF_OM_; *n* = 4) and nutrients in feces (protein, ash, Ca and P; *n* = 4) using sparse partial least square-discriminant analysis showing different enrichment between the treatment groups for **(A)** component 1 and **(B)** component 2. Con, control diet; LA, diet containing lactic acid- treated cereals; Con-Phy, diet with phytase supplementation; and LA-Phy, diet with phytase and lactic acid-treated cereals.

**FIGURE 3:**
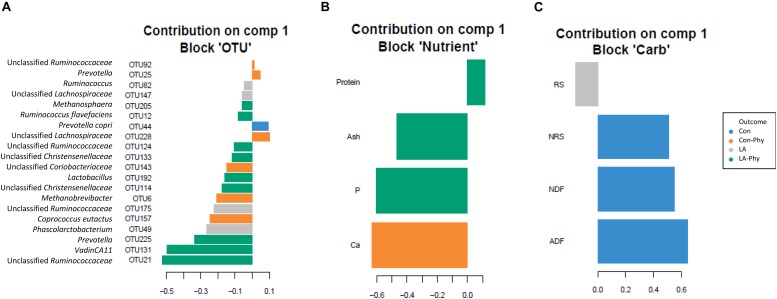
Loading plots of sparse partial least square-discriminant analysis showing the best discriminant bacterial operational taxonomic units (OTU; *n* = 20; relative OTU abundance >0.04%) and complex carbohydrates (RS, NRS, aNDF_OM_ and ADF_OM_; *n* = 4) or nutrients in feces (protein, ash, Ca and P; *n* = 4). The sPLS-DA identified the best discriminant bacterial OTUs on component 1 at more than 0.04% relative abundance of all **(A)** OTUs, **(B)** nutrients and **(C)** carbohydrates (method = “median”). Colors indicate the treatment groups where the mean abundance of the OTUs, nutrients and carbohydrates is maximal. Con, control diet; LA, diet containing lactic acid- treated cereals; Con-Phy, diet with phytase supplementation; and LA-Phy, diet with phytase and lactic acid-treated cereals.

**FIGURE 4:**
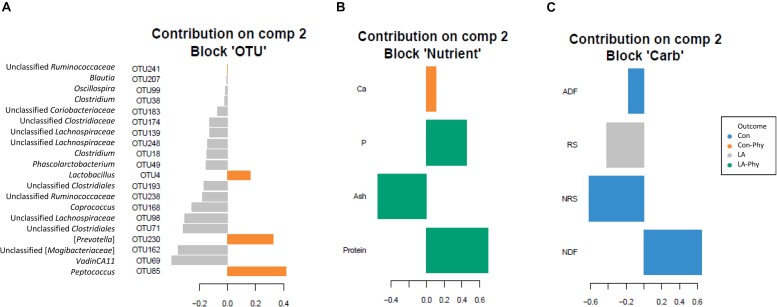
Loading plots of sparse partial least square-discriminant analysis showing **(A)** the best discriminant bacterial operational taxonomic units (OTU; *n* = 20; relative OTU abundance >0.04%) and **(B)** complex carbohydrates (RS, NRS, aNDF_OM_ and ADF_OM_; *n* = 4) or **(C)** nutrients in feces (protein, ash, Ca and P; *n* = 4). Colors indicate the treatment groups where the mean abundance of the OTUs, nutrients and carbohydrates is maximal. Con, control diet; LA, diet containing lactic acid- treated cereals; Con-Phy, diet with phytase supplementation; and LA-Phy, diet with phytase and lactic acid-treated cereals.

**FIGURE 5:**
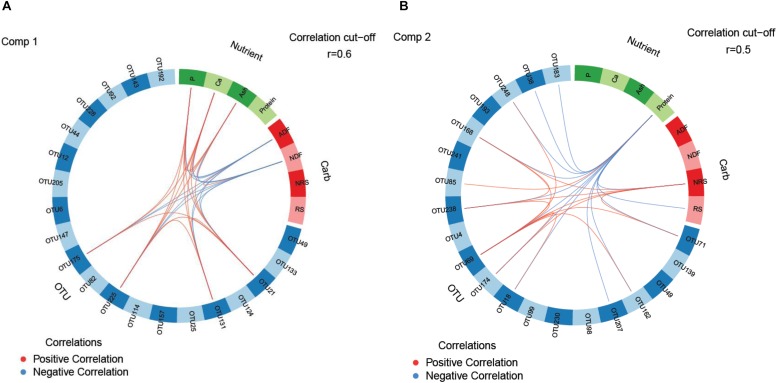
The circos plots of sparse partial least square-discriminant analysis displaying correlations between the identified best discriminant bacterial operational taxonomic units (OTU; *n* = 20; relative OTU abundance >0.04%), complex carbohydrates (RS, NRS, aNDF_OM_ and ADF_OM_; *n* = 4) and nutrients in feces (protein, ash, Ca and P; *n* = 4) for **(A)** component 1 and **(B)** component 2. Positive and negative correlations (| r| > 0.6 for component 1 and | r| > 0.5 for component 2) are displayed by red and blue links, respectively. OTU4 *Lactobacillus*; OTU6 *Methanobrevibacter*; OTU12 *Ruminococcus flavefaciens*; OTU18 *Clostridium*; OTU21 *Ruminococcaceae*; OTU25 *Prevotella*; OTU38 *Clostridium*; OTU44 *Prevotella copri*; OTU49 *Phascolarctobacterium*; OTU69 *VadinCA11*; OTU71 Unclassified *Clostridiales*; OTU82 *Ruminococcus*; OTU85 *Peptococcus*; OTU92 Unclassified *Ruminococcaceae*; OTU98 Unclassified *Lachnospiraceae*; OTU99 *Oscillospira*; OTU114 Unclassified *Christensenellaceae*; OTU124 Unclassified *Ruminococcaceae*; OTU131 *VadinCA11*; OTU133 Unclassified *Christensenellaceae*; OTU139 Unclassified *Lachnospiraceae*; OTU143 Unclassified *Coriobacteriaceae*; OTU147 Unclassified *Lachnospiraceae*; OTU157 *Coprococcus eutactus*; OTU162 Unclassified [*Mogibacteriaceae*]; OTU168 *Coprococcus*; OTU174 Unclassified *Clostridiaceae*; OTU175 Unclassified *Ruminococcaceae*; OTU183 Unclassified *Coriobacteriaceae*; OTU192 *Lactobacillus*; OTU193 Unclassified *Clostridiales*; OTU205 *Methanosphaera*; OTU207 *Blautia*; OTU225 *Prevotella*; OTU228 Unclassified *Lachnospiraceae*; OTU230 [*Prevotella*]; OTU238 Unclassified *Ruminococcaceae*; OTU241 Unclassified *Ruminococcaceae*; OTU248 Unclassified *Lachnospiraceae.*

Supervised sparse partial least squares-discriminant analysis (sPLS-DA) was used to identify the most influential OTUs, and their relation to protein, ash, Ca, and P as well as complex carbohydrate composition (aNDF_OM_, ADF_OM_, RS, and NRS) in feces for component 1 and 2. The most influential OTUs and chemical components were visualized in heatmaps (Figures 2A,B) and loading plots (Figures 3, 4). The strongest correlations among the best 20 discriminant OTUs, complex carbohydrates and other nutrients in feces are displayed in circos plots for component 1 (Figure 5A) and component 2 (Figure 5B), respectively. For component 1 (Figure 5A), four OTUs (unclassified *Ruminococcaceae*-OTU21, *VadinCA11*-OTU131, unclassified *Ruminococcaceae*-OTU175, and *Prevotella*-OTU225) were positively linked to P and Ca, whereby unclassified *Ruminococcaceae*-OTU21 and *Prevotella*-OTU225 were additionally positively correlated to fecal ash. In contrast, these four OTUs were negatively correlated to ADF_OM_ and aNDF_OM_ in feces. Moreover, P, Ca, and ash correlated negatively to ADF_OM_ and aNDF_OM_. For component 2 (Figure 5B), only *Peptococcus*-OTU85 was positively linked to protein, whereas 11 OTUs (*Clostridium*-OTU18 and -OTU38, *VadinCA11*-OTU69, unclassified *Clostridiales*-OTU71, unclassified [*Mogibacteriaceae*]-OTU162, *Coprococcus*-OTU168, unclassified *Clostridiaceae*-OTU174, unclassified *Coriobacteriaceae*-OTU183, *Blautia*-OTU207, unclassified *Ruminococcaceae*-OTU238, and unclassified *Lachnospiraceae*-OTU248) were negatively correlated with fecal protein. Additionally, *VadinCA11*-OTU69, unclassified *Clostridiales*-OTU71, *Coprococcus*-OTU168, unclassified *Clostridiaceae*-OTU174 and unclassified *Ruminococcaceae*-OTU238 also positively correlated to NRS in feces. Moreover, the protein in feces were negatively linked with RS and NRS in feces.

## Discussion

Albeit not being representative for the bacterial community in the small intestine and proximal large intestine, the fecal microbiota of pigs is qualitatively similar and hence representative for the bacterial microbiome composition in the more distal parts of the large intestine (Zhao et al., 2015). Therefore, characterization of the bacterial community in feces is valid and informative to our understanding of nutrient-microbiota interactions, contributing to the discovery of cause-and-effect relationships between dietary changes and expression and shedding of virulence factor genes of common opportunistic pathogens in pigs. Due to the present manipulation of the intestinal P availability by both phytase supplementation and pre-treatment of the cereals with 2.5% LA together with changes in the complex carbohydrate fraction due to the LA-treatment of grains (Metzler-Zebeli et al., 2014; Metzler-Zebeli et al., 2015), we predicted changes mainly in the proteolytic and fibrolytic fecal bacterial communities as previously observed in pigs and ruminants (Metzler-Zebeli et al., 2014; Metzler-Zebeli et al., 2015; Metzler-Zebeli et al., 2018). The present results showed that both dietary treatments caused distinct community composition profiles of the metabolically active bacteria in feces of pigs; however, without affecting total bacterial gene copy numbers or virulence factor expression of *E. coli* and *C. perfringens*. Notably, all major bacterial families were either affected by the dietary phytase or LA-treated cereals, whereby more taxa were modulated by the LA-treated cereals than by the phytase supplementation. This was reflected in the α-diversity analysis showing that only the LA treatment of cereals impacted species richness (Chao1) and evenness (Shannon). However, as we did not observe an increase in opportunistic pathogens, this decrease in diversity may not have weakened the stability of the bacterial community in the distal large intestine. We could associate several bacterial OTUs with the fecal nutrient concentrations, showing that the bacterial community adapted rapidly to changes in nutrient availabilities in the distal large intestine. Digestion and fermentation continuously advance throughout the digestive tract; therefore, bacterial abundances observed in feces are a consequence of alterations in the digestive and fermentative processes occurring proximally. Part of the observed dietary effects may be therefore linked to subsequent alterations in the microbe-microbe-interactions and cross-feeding of primary fermentation metabolites throughout the lower intestine (Flint et al., 2015). This relationship was supported by the present correlations between the most discriminant OTUs and fecal concentrations of Ca, P, protein and complex carbohydrate fractions.

While the LA treatment of cereals released the phytate-bound phosphate groups pre-feeding (Metzler-Zebeli et al., 2014; Vötterl et al., 2018), the supplemental phytase was only activated in the stomach when reaching their pH optima (Zhang et al., 2010). Although the chemical composition of feces can only be indicative for changes in intestinal absorption, present results suggest that the phytase was more efficient to improve the intestinal P availability than the LA treatment of cereal grains, whereby combining dietary phytase and LA-treated cereals acted synergistically on reducing the fecal P content. Notably, fecal Ca was not similarly affected by both treatments as indicated by the varying Ca:P ratio among the four treatment groups. Indeed, the Phy × LA interaction for the Ca:P ratio suggested that more Ca was retained in large intestinal digesta and feces with the combination of dietary phytase and LA-treated cereals, which may have been due to buffering properties of Ca, binding to dietary residuals or bacterial incorporation (Metzler and Mosenthin, 2008). Feces became enriched with aNDF_OM_ and ADF_OM_ with the LA-treated cereals, indicating low fermentability of the residual fiber fractions. Since feces also contained more NRS, changes in gut motility and a subsequently higher intestinal passage may be a possible explanation for these observations, modifying the time available for bacterial substrate utilization and proliferation (Le Goff et al., 2002; Van Leeuwen et al., 2006; Wilfart et al., 2007; Metzler-Zebeli et al., 2019). Therefore, it suggests the fecal transit time and available substrates are considered as vital factors influencing bacterial substrate utilization and proliferation, especially enteric pathogens. This may be additionally supported by the high fecal DM content in pigs fed the LA-treated cereals, which may contribute to reducing the risk of diarrhea as more water is absorbed.

Both dietary phytase supplementation and LA-treatment of grains led to dramatic alterations in the dominant families, all belonging to the *Firmicutes* phylum. The main beneficiary of the community shifts was the family *Clostridiaceae*, becoming especially enriched in feces when both treatments were combined. This diverse and versatile family comprises a plethora of metabolic capabilities including starch-degrading, fibrolytic and proteolytic capacities as well as many important porcine enteric pathogens, e.g., *C. perfringens*. Against this background, the limited classification of many *Clostridiaceae* genera and species makes it difficult to predict their metabolic dependencies within the present bacterial communities and their role for host health. Correlations displayed in the circos plots indicated negative relationships of *Clostridium*-OTU18 and -OTU38, both representing species related to *C. saccharolyticum*, and fecal protein. *C. saccharolyticum* mainly utilizes di- and oligosaccharides (Murray et al., 1982). Their fecal enrichment may have been therefore an indirect result of cross-feeding of sugars released by other hemicellulolytic and cellulolytic species. Similar metabolic preferences may explain the negative relationships between dietary protein and other best discriminant OTUs observed in the circos plots. This assumption is supported by the positive link of *Coprococcus*-OTU168, unclassified *Ruminococcaceae*-OTU238, unclassified *Clostridiales*-OTU71 and *VacinCA11*-OTU69 with NRS, indicating amylolytic capabilities.

The importance of intestinal P availability for bacterial proliferation (Metzler and Mosenthin, 2008) was indicated by the decline of the predominant *Lactobacillus* and lesser abundant butyrate-producing *Dorea* in feces, both saccharolytic taxa, after feeding pigs the phytase-containing diets. Previous findings in pigs (Metzler-Zebeli et al., 2010) and rats (Ten Bruggencate et al., 2004) showed that the intestinal abundance of the *Lactobacillus* group may be rather related to the Ca than to the P availability in digesta. This may have been also the case in the present study as the dietary phytase supplementation reduced both the fecal Ca and P content. Several underlying modes of action may explain this finding and may be related to buffering properties of the insoluble Ca-P complex, which forms in the intestinal lumen at pH values above 5 (Govers and Van der Meer, 1993) and precipitates for bacterial cells cytotoxic fatty and bile acids (Kurdi et al., 2006). Also feasible is a modification in the mucosal attachment of *Lactobacillus* due to a variation in free Ca ions as shown *in vitro* (Lim and Ahn, 2012). Other Ca- or P-related dependencies in bacterial metabolism and enzyme activation were also likely but it requires a more functional approach (e.g., metatranscriptomics) to predict the affected metabolic pathways. Although *Lactobacillus*- and *Dorea*-OTUs were not among the most influential OTUs linked to fecal Ca and P, the sPLS-DA identified *Prevotella copri*-related OTU225, two *Ruminococcaceae*-OTUs (OTU21 and OTU175) and OTU131 belonging to the archaeal *VadinCA11* group to have depended on the fecal Ca and P concentration. Since the methanogenic *VadinCA11*-OTU131 was also positively linked to *Prevotella*-OTU225, this may indicate cross-feeding of hydrogen which stimulated their abundance rather than the fecal Ca and P availability. Additionally, the inverse relationship of these most discriminant OTUs and fecal Ca and P with fecal aNDF_OM_ and ADF_OM_ concentrations may support depression in bacterial fiber degradation with lower Ca and P availability in the large intestine as has been reported for rumen bacteria under low rumen P availability (Harder et al., 2015).

Despite an enrichment of feces with aNDF_OM_ and, as trends, starch fractions, the decline in the fecal abundances of *Prevotella*, *Lachnospiraceae* and *Ruminococcaceae*, which comprise many amylolytic, pullulanolytic and fibrolytic species (Kraler et al., 2016; La Reau et al., 2016), with the LA-treated cereals may have indicated that their preferred substrate became depleted in the distal large intestine. With substrate availability being a major factor for population dynamics (Kelly et al., 2017), the lower proportional RS contribution to total starch in feces of pigs fed the LA-treated grains would support this assumption. Fittingly, the circos plot for component 2 illustrated positive relationships between the NRS content and saccharolytic bacteria, such as *Coprococcus*, *Lachnospiraceae*-, *Ruminococcaceae-* and *Clostridium saccharobutylicum*-like OTUs, and methanogens of the *VadinCA11* group. In addition, it is feasible that the fecal composition of aNDF_OM_ differed due to the LA treatment (Metzler-Zebeli et al., 2014) which was potentiated by the progressing digestion and fermentation throughout the large intestine, thereby contributing to the lower fecal abundances of *Prevotella*, *Lachnospiraceae* and *Ruminococcaceae*.

Previously, we found a positive relationship between increased dietary Ca and P levels and the intestinal abundance of proteolytic bacterial groups including *Clostridium* cluster I, *Enterobacteriaceae*, *Campylobacter* and *Helicobacter* (Metzler-Zebeli et al., 2013). Although these taxa are considered commensals in the gastrointestinal tract of pigs, they also comprise important opportunistic pathogens. While being only a tendency for a difference, present results confirmed our hypothesis and supported the previously reported link between intestinal P availability and *Enterobacteriaceae* numbers (Metzler-Zebeli et al., 2013). Feeding the LA-treated grains also decreased the fecal P content. Nevertheless, the P in digesta of the distal large intestine with the LA-treated cereals was obviously sufficient to support proliferation of *Enterobacteriaceae*, *Campylobacteraceae* and *Helicobacteraceae*. Results for *E. coli* virulence factor gene expression showed that enterotoxigenic and shiga-toxin-producing *E. coli* were present but equally expressed their virulence genes among pig groups. Despite the predominance of the whole *Clostridiaceae* family, expression of *cpa* of *C. perfringens* was not detectable. Therefore, it may be assumed that despite the present changes in the bacterial communities including the reduced bacterial diversity with the LA-treated cereals, this did not lead to temporal instability and depletion of distinct commensal species that suppress virulence factor gene expression, resulting in a more colitogenic commensal composition (Frosali et al., 2015). The greatest effect that we found on opportunistic pathogens was the rise in *Erysipelotrichaceae* with the LA-treated cereals. However, this increase appeared to be related to other nutrients than P as phytase supplementation did not modify their abundance.

The present results demonstrate that both dietary phytase supplementation and LA-treatment of cereals caused specific alterations in the fecal viable bacterial community composition, whereas only the LA-treated grains reduced the bacterial diversity. Although not altered by the present dietary treatments, virulence factor expression in feces has implications for health risk assessment to reduce disease transmission within a pig herd and spreading of foodborne zoonotic diseases. The sPLS-DA further allowed the characterization of bacterial nutrient dependencies in the large intestine, indicating a link between the P availability and complex carbohydrate composition in feces and alterations in the predominant genera belonging to *Clostridiaceae*, *Lactobacillaceae*, *Lachnospiraceae*, *Ruminococcaceae* and *Prevotellaceae*.

## Data availability statement

The datasets generated for this study can be found in the NCBI Bioproject databank (PRJNA522345).

## Ethics statement

The animal study was reviewed and approved by the Institutional Ethics Committee of the University of Veterinary Medicine Vienna (Vienna, Austria) and the national authority according to paragraph 8 of the Law for Animal Experiments, Tierversuchsgesetz (TVG) (BMWFW-68.205/0158-WF/V/3b/2016).

## Author Contributions

BM-Z and QZ conceived and designed the experiments. JK, JV, and BM-Z performed the experiments. JK and BM-Z analyzed the data and wrote the manuscript. BM-Z edited and finalized the manuscript. All authors read and approved the final version of the manuscript.

## Conflict of Interest

The authors declare that the research was conducted in the absence of any commercial or financial relationships that could be construed as a potential conflict of interest.

## Footnotes

1 www.ncbi.nlm.nih.gov/tools/primer-blast/

2 https://cutadapt.readthedocs.org/

3 https://blast.ncbi.nlm.nih.gov/

## Abbreviations

ADF_OM_: acid detergent fiber exclusive of residual ash
aNDF_OM_: alpha amylase-stable neutral detergent fiber exclusive of residual ash
Ca: calcium
Con: control diet
Con-Phy: control diet with 500 FTU/kg phytase
*Cpa*: *Clostridium perfringens* alpha toxin
DM: dry matter
LA: diet containing lactic acid treated cereals
LA-Phy: diet with 500 FTU/kg phytase and lactic acid-treated cereals
NRS: non-resistant starch
OTU: operational taxonomic units
P: phosphorus
RS: resistant starch
*STa*: heat-stable enterotoxin of enterotoxigenic *Escherichia coli*
*Stx2e*: shiga toxin type 2e of shiga-toxin producing *E. coli*.
