# Supplementary material for: Lactic Acid Treatment of Cereals and Dietary Phytase Modified Fecal Microbiome Composition Without Affecting Expression of Virulence Factor Genes in Growing Pigs
Source: Front Microbiol. 2019 Oct 15;10:2345. doi: 10.3389/fmicb.2019.02345 (PMC6808178; doi:10.3389/fmicb.2019.02345)
Supplement: Supplementary file 1 [file Data_Sheet_1.PDF]

Running title: Lactic acid treated cereals, phytase and fecal microbiome

Lactic Acid Treatment of Cereals and Dietary Phytase Modified Fecal Microbiome Composition without Affecting Expression of Virulence Factor Genes in Growing Pigs

Jutamat Klinsoda[1,2], Julia Vötterl[1], Qendrim Zebeli[1] and Barbara U. Metzler-Zebeli[1]*

[1]Institute of Animal Nutrition and Functional Plant Compounds, University of Veterinary Medicine, Vienna, Austria
[2] Institute of Food Research and Product Development, University of Kasetsart, Bangkok, Thailand.

* Correspondence:
Barbara U. Metzler-Zebeli
Barbara.Metzler@vetmeduni.ac.at

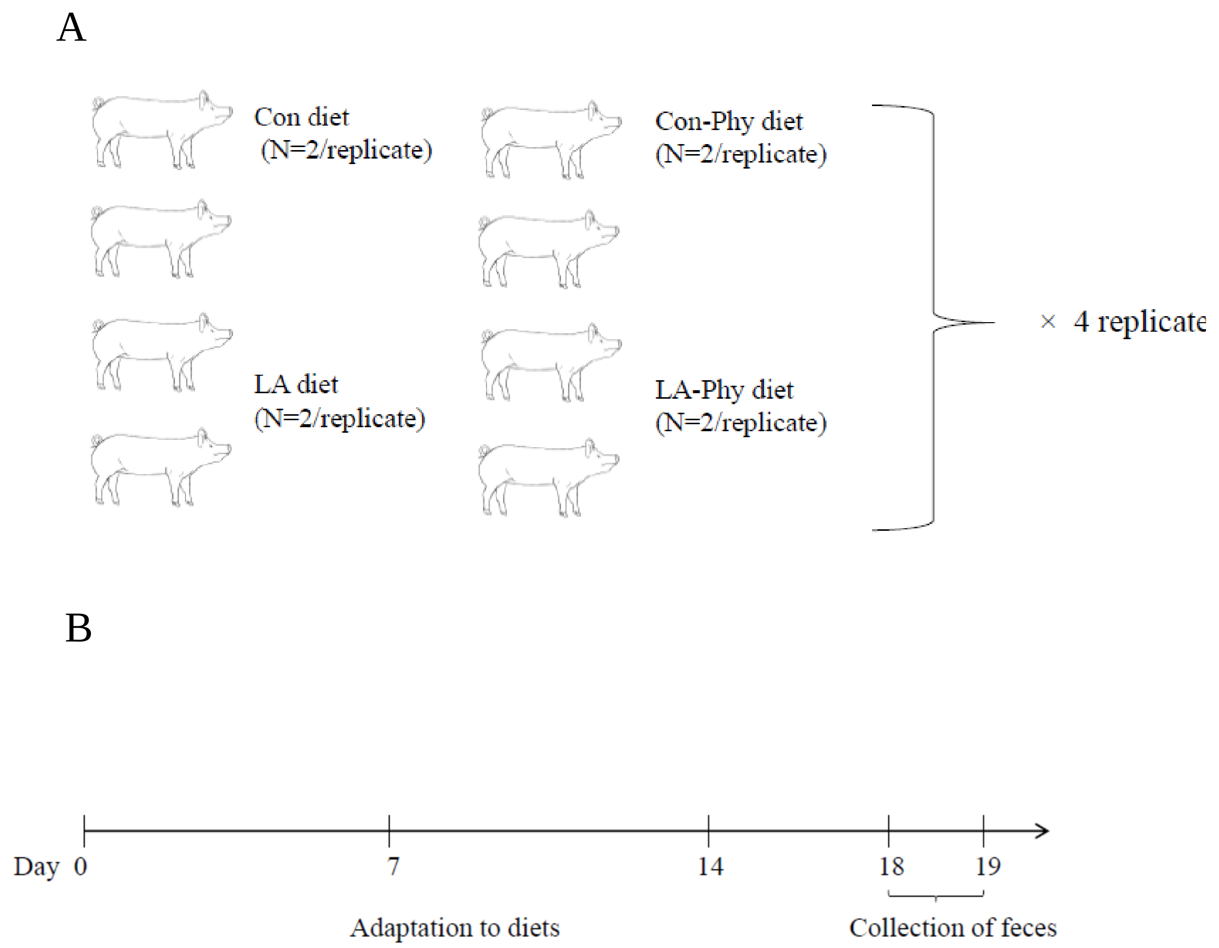

Figure S1 A) Experimental design; and B) experimental schedule.

TABLE S1 Oligonucleotide primers used for quantitative PCR

**Bacteria group**	**Primer sequence (5' to 3')**	**PCR Efficiency**	R^2	**Amplicon size (bp)**	**Reference**
Universal primer	F:CCTACGGGAGGCAGCAG<br>R:ATTACCGCGGCTG CTGG	1.94	1.00	189	Muyzer et al., 1993
cpa	F:AAGAACTAGTAGCTTACATATCAACTAGTGGTG<br>R:TTTCCTGGGTTGTCCATTTCC	1.98	0.99	124	Schlegel et al., 2012
ETEC *STa*	F:ATGAAAAAGCTAATGTTGGC<br>R:TACAACAAAGTTCACAGCAG	2.01	0.99	193	Metzler-Zebeli et al., 2013
STEC *Stx2e*	F:ATGAAGAAGATGTTTATAGCG<br>R:TCAGTTAAACTTCACCTGGGC	1.94	1.00	264	Metzler-Zebeli et al., 2013

Cpa, *Clostridium perfringens* alpha toxin; STa, heat-stable enterotoxin of enterotoxigenic *Escherichia coli*; Stx2e, Shiga toxin type 2e of shiga-toxin producing *E. coli*.

TABLE S2 BLAST search results for selected operational taxonomic units (OTU) *

OTU	Best BLAST hit[†]	Accession Number	E-Value	Percent Identity
4	*Lactobacillus antri*	NZ_GG700732.1	2E-40	100%
6	*Methanobrevibacter millerae*	NZ_CP011266.1	2E-33	100%
12	*Ruminococcus flavefaciens*	NZ_JNKE01000007.1	2E-45	94%
14	*Lactobacillus pontis*	NZ_AZGO01000009.1	9E-154	99%
18	*Clostridium saccharobutylicum*	NC_022571.1	2E-45	94%
19	*Butyricicoccus pullicaecorum*	NZ_KB976106.1	8E-164	93%
21	*Ruminococcaceae bacterium*	NZ_FWXP01000018.1	1E-32	99%
23	*Bariatricus massiliensis*	NZ_LT574835.1	7E-45	94%
25	*Prevotella stercorea*	NZ_JH379355.1	5E-36	97%
27	*Eubacterium xylanophilum*	NZ_JAEB01000051.1	1E-41	93%
34	[*Clostridium*] *viride*	NZ_JHZO01000008.1	4E-147	91%
35	*Intestinibacter bartlettii*	NZ_DS499568.1	0	97%
36	*Eubacterium cylindroides*	NC_021019.1	2E-40	100%
38	*Clostridium saccharobutylicum*	NC_022571.1	2E-45	94%
39	*Lactobacillus panis*	NZ_AZGM01000084.1	2E-40	100%
41	*Collinsella bouchesdurhonensis*	NZ_LT699736.1	7E-45	94%
44	*Prevotella copri*	NZ_GG703856.1	2E-39	99%
49	*Phascolarctobacterium succinatutens*	NZ_GL830850.1	2E-40	100%
55	*Prevotella baroniae*	NZ_AUFQ01000046.1	2E-39	99%
58	*Clostridium celatum*	NZ_KB291630.1	2E-44	94%
60	*Coprococcus comes*	NZ_GG662006.1	0	98%
66	*Eubacterium xylanophilum*	NZ_JAEB01000051.1	2E-45	94%
69	Unclassified			
70	*Dialister succinatiphilus*	NZ_JH591188.1	3E-38	99%
71	*Blautia hansenii*	NZ_GG698591.1	1E-41	93%
82	*Ruminococcus champanellensis*	NC_021039.1	1E-157	92%
85	*Desulfotomaculum hydrothermale*	NZ_CAOS01000011.1	7E-125	87%
92	*Ruminococcus flavefaciens*	NZ_JAEF01000027.1	4E-32	95%
93	*Dorea formicigenerans*	NZ_AAXA02000016.1	0	98%
94	*Ruminococcus lactaris*	NZ_DS990170.1	2E-174	95%
95	*Anaerobium acetethylicum*	NZ_FMKA01000073.1	5E-166	94%

98	[*Clostridium*] *symbiosum*	NZ_GL834329.1	7E-40	92%
99	*Clostridium phoceensis*	NZ_LN866274.1	1E-171	94%
102	*Eubacterium xylanophilum*	NZ_JAEB01000051.1	2E-45	94%
114	*Clostridium botulinum*	NC_009698.1	4E-142	90%
123	[*Eubacterium*] *cellulosolvens*	NZ_CM001487.1	4E-167	94%
124	*Eubacterium coprostanoligenes*	NZ_FUWW01000033.1	3E-143	90%
125	*Stomatobaculum longum*	NZ_JH590865.1	3E-138	90%
131	*Methanogenic archaeon*	NZ_CP014214.1	2E-164	95%
133	*Christensenella sp. Marseille*	NZ_LT700187.1	1E-131	88%
134	*Ruminococcus bicirculans*	NZ_HF545617.1	1E-166	94%
139	*Fusicatenibacter saccharivorans*	NZ_CYYV01000001.1	2.00E-175	95%
143	*Adlercreutzia equolifaciens*	NC_022567.1	9E-154	92%
144	*Blautia obeum*	NZ_DS264339.1	0	96%
147	[*Clostridium*] *asparagiforme*	NZ_GG657593.1	0	97%
150	*Olsenella scatoligenes*	NZ_LOJF01000014.1	2E-155	92%
155	*Clostridium disporicum*	NZ_CYZX01000047.1	0	97%
157	*Coprococcus eutactus*	NZ_DS483539.1	0	98%
161	*Clostridium disporicum*	NZ_CYZX01000047.1	0	97%
162	*Mobilibacterium timonense*	NZ_FUHN01000006.1	2E-134	89%
168	*Anaerostipes hadrus*	NZ_CP012098.1	0	96%
174	*Clostridium butyricum*	NZ_CP013252.1	0	97%
175	*Ruminococcus flavefaciens*	NZ_JAEF01000027.1	9E-114	86%
176	*Selenomonas ruminantium*	NZ_FNCM01000034.1	7E-135	96%
178	*Ruminococcus faecis*	NZ_BBDW01000030.1	0	97%
183	*Olsenella scatoligenes*	NZ_LOJF01000014.1	0	96%
192	*Lactobacillus gigeriorum*	NZ_AYZO01000082.1	2E-144	98%
193	*Intestinibacter bartlettii*	NZ_DS499553.1	8E-179	96%
197	*Blautia obeum*	NZ_CYZA01000043.1	2E-180	96%
205	*Methanosphaera stadtmanae*	NC_007681.1	3E-163	94%
207	*Blautia hansenii*	NZ_GG698591.1	4E-177	95%
222	*Megasphaera elsdenii*	NZ_CP009240.1	3E-153	99%
225	*Prevotella copri*	NZ_GG703854.1	3E-109	91%
227	*Intestinibacillus massiliensis*	NZ_LT707042.1	1E-172	95%
228	[*Clostridium*] *saccharolyticum*	NZ_NFKU01000053.1	0	96%

230	*Prevotellamassilia timonensis*	NZ_LT629844.1	1E-126	88%
236	*Lactobacillus pontis*	NZ_AZGO01000009.1	9E-149	99%
238	*Oscillibacter ruminantium*	NZ_DF158903.1	1E-151	91%
240	*Brevibacterium album*	NZ_AUFJ01000004.1	1E-16	100%
241	*Eubacterium coprostanoligenes*	NZ_FUWW01000033.1	5E-146	91%
248	*Eubacterium ramulus*	NZ_KI271105.1	2E-175	95%
254	*Eubacterium brachy*	NZ_KI535272.1	1E-142	90%
260	[*Ruminococcus*] *gnavus*	NZ_JAGQ01000002.1	1E-167	94%
264	*Coprococcus comes*	NZ_GG662006.1	0	98%
267	*Eubacterium sulci*	NZ_CP012068.1	1E-146	91%
269	*Anaerotruncus colihominis*	NZ_DS544191.1	9E-139	90%
270	*Ruminococcus lactaris*	NZ_DS990170.1	2.00E-159	93%
271	*Phocea massiliensis*	NZ_LT629937.1	2.00E-144	90%
280	*Prevotella oris*	NZ_BAJC01000066.1	2E-116	86%
281	*Prevotella oris*	NZ_BAJC01000066.1	7E-125	87%
284	[*Eubacterium*] *hallii*	NZ_ACEP01000116.1	0	98%
293	*Blautia hansenii*	NZ_GG698591.1	0	96%
298	*Ruminococcus lactaris*	NZ_DS990170.1	9E-169	94%
301	*Eubacterium xylanophilum*	NZ_JAEB01000051.1	3E-178	96%
303	*Eubacterium sulci*	NZ_CP012068.1	5E-136	89%
305	*Methanogenic archaeon*	NZ_CP014214.1	7E-115	87%
306	*Methanogenic archaeon*	NZ_CP014214.1	2E-124	88%
307	*Acetivibrio ethanolgignens*	NZ_LNAM01000152.1	2E-170	94%
315	*Campylobacter hyointestinalis subsp. lawsonii*	NZ_CP015576.1	0	97%
318	*Butyricicoccus pullicaecorum*	NZ_KB976105.1	9E-154	92%
319	*Eubacterium xylanophilum*	NZ_JAEB01000051.1	5E-156	92%
329	*Sporobacter termitidis*	NZ_FQXV01000034.1	8E-164	93%
331	*Blautia obeum*	NZ_CYZA01000043.1	2E-175	95%
348	*Eubacterium ramulus*	NZ_KI271105.1	2E-179	96%
362	*Emergencia timonensis*	NZ_FLKM01000009.1	1E-141	90%
368	*Bulleidia extructa*	NZ_ADFR01000011.1	9E-119	93%
370	*Pseudoflavonifractor capillosus*	NZ_AAXG02000048.1	3E-148	91%
371	*Thermincola ferriacetica*	NZ_LGTE01000045.1	4E-112	86%

375	*Clostridium disporicum*	NZ_CYZX01000047.1	0	97%
377	[*Ruminococcus*] *gnavus*	NZ_JAGQ01000002.1	2E-169	94%
380	[*Clostridium*] *saccharolyticum*	NC_014376.1	5E-166	94%
382	[*Clostridium*] *viride*	NZ_JHZO01000008.1	9E-149	91%
387	*Clostridium polynesiense*	NZ_CCXI01000161.1	4E-127	88%
389	[*Clostridium*] *asparagiforme*	NZ_GG657593.1	0	96%
395	*Eubacterium sulci*	NZ_CP012068.1	3E-148	91%
400	*Clostridium disporicum*	NZ_CYZX01000047.1	0	97%
413	[*Clostridium*] *saccharolyticum*	NC_014376.1	0	96%
416	*Terrisporobacter glycolicus*	NZ_AUUB01000009.1	0	97%
417	*Oscillibacter valericigenes*	NC_016048.1	7E-165	93%
419	*Olsenella profusa*	NZ_AWEZ01000030.1	5E-141	90%
420	*Clostridium saccharoperbutylacetonicum*	NC_020291.1	0	96%
423	*Blautia obeum*	NZ_DS264339.1	8E-174	95%
442	*Coprococcus comes*	NZ_GG662006.1	0	97%
443	[*Ruminococcus*] *gnavus*	NZ_JAGQ01000002.1	1E-172	95%
444	*Blautia obeum*	NZ_CYZA01000043.1	0	98%
446	*Slackia piriformis*	NZ_JH815198.1	2E-145	91%
449	*Fusicatenibacter saccharivorans*	NZ_CYYV01000001.1	2E-170	94%
458	*Mailhella massiliensis*	NZ_LT706945.1	4E-152	99%
460	*Clostridium disporicum*	NZ_CYZX01000047.1	0	97%
462	*Lactobacillus coleohominis*	NZ_GG698808.1	4E-152	99%
467	*Butyricicoccus pullicaecorum*	NZ_KB976106.1	4E-152	91%
474	*Clostridium phoceensis*	NZ_LN866274.1	7E-160	93%
484	[*Clostridium*] *saccharolyticum*	NZ_NFKU01000053.1	1E-172	95%
485	*Blautia obeum*	NZ_DS264339.1	2E-170	94%
489	*Provencibacterium massiliense*	NZ_FWCH01000011.1	3E-153	92%
492	*Mitsuokella multacida*	NZ_GG697141.2	3E-143	98%
495	*Coprococcus comes*	NZ_GG662006.1	0	96%
510	*Ruminococcus lactaris*	NZ_DS990170.1	5E-156	92%
513	*Slackia exigua*	NZ_GG700630.1	9E-134	89%
516	[*Ruminococcus*] *gnavus*	NZ_JAGQ01000002.1	2E-175	95%
517	*Ruminococcus bicirculans*	NZ_HF545617.1	3E-163	93%

519	[*Bacteroides*] *pectinophilus*	NZ_DS996921.1	2E-159	93%
524	*Clostridium phoceensis*	NZ_LN866274.1	6E-170	94%
535	*Blautia obeum*	NZ_DS264339.1	8E-164	93%
536	*Clostridium disporicum*	NZ_CYZX01000047.1	0	97%
540	*Ruminococcus lactaris*	NZ_DS990170.1	1E-137	90%
547	*Papillibacter cinnamivorans*	NZ_FWXW01000005.1	7E-145	90%
553	[*Bacteroides*] *pectinophilus*	NZ_DS996921.1	5E-156	92%
557	*Lactobacillus antri*	NZ_GG700732.1	9E-154	99%
568	*Clostridium disporicum*	NZ_CYZX01000047.1	0	97%
575	*Terrisporobacter glycolicus*	NZ_AUUB01000009.1	0	97%
580	[*Clostridium*] *saccharolyticum*	NZ_NFKU01000053.1	2E-179	96%
581	*Mitsuokella multacida*	NZ_GG697141.2	7E-145	98%
588	*Ruminococcus lactaris*	NZ_DS990170.1	4E-172	95%
590	*Ruminococcus lactaris*	NZ_DS990170.1	1E-172	95%
594	*Eubacterium xylanophilum*	NZ_JAEB01000051.1	0	96%
600	*Sporobacter termitidis*	NZ_FQXV01000034.1	3E-148	91%
608	*Lactobacillus pontis*	NZ_AZGO01000009.1	9E-154	99%
610	*Clostridium disporicum*	NZ_CYZX01000047.1	0	97%
619	*Coprococcus comes*	NZ_GG662006.1	5E-176	95%
620	*Ruminococcus champanellensis*	NC_021039.1	2E-160	93%
623	*Blautia obeum*	NZ_DS264339.1	8E-164	93%
630	*Lactobacillus pontis*	NZ_AZGO01000009.1	9E-154	99%
640	*Anaerobium acetethylicum*	NZ_FMKA01000073.1	2E-179	96%
660	*Ruminococcus lactaris*	NZ_DS990170.1	2E-180	96%
661	*Sporobacter termitidis*	NZ_FQXV01000034.1	2E-170	94%
662	*Pseudoflavonifractor capillosus*	NZ_AAXG02000048.1	6E-175	95%
669	*Papillibacter cinnamivorans*	NZ_FWXW01000005.1	1E-131	88%
680	[*Eubacterium*] *hallii*	NZ_ACEP01000116.1	0	98%
690	*Intestinibacillus massiliensis*	NZ_LT707042.1	3E-148	91%
691	*Desulfotomaculum hydrothermale*	NZ_CAOS01000011.1	2E-124	87%
704	*Clostridium autoethanogenum*	NC_022592.1	2E-116	86%
720	*Clostridium disporicum*	NZ_CYZX01000047.1	5E-166	94%
728	*Eubacterium xylanophilum*	NZ_JAEB01000051.1	3E-178	96%
729	*Massilioclostridium coli*	NZ_FMIZ01000006.1	2E-164	93%

735	*Blautia obeum*	NZ_DS264339.1	4E-177	95%
741	*Terrisporobacter glycolicus*	NZ_AUUB01000009.1	0	97%
747	[*Eubacterium*] *hallii*	NZ_ACEP01000116.1	3E-173	96%
748	*Phascolarctobacterium succinatutens*	NZ_GL830850.1	1E-131	95%
761	*Papillibacter cinnamivorans*	NZ_FWXW01000005.1	3E-168	94%
779	*Blautia obeum*	NZ_DS264339.1	4E-172	95%
789	*Mitsuokella multacida*	NZ_GG697141.2	1E-141	97%
797	*Blautia obeum*	NZ_DS264339.1	8E-174	95%
820	*Eubacterium oxidoreducens*	NZ_FMXR01000001.1	2E-175	95%
822	*Blautia hansenii*	NZ_GG698591.1	4E-177	95%
829	*Butyricicoccus pullicaecorum*	NZ_KB976106.1	4E-137	89%
835	*Anaerostipes hadrus*	NZ_CP012098.1	0	98%
837	*Eubacterium xylanophilum*	NZ_JAEB01000051.1	5E-171	94%
848	*Ruminococcus lactaris*	NZ_DS990170.1	2E-174	95%
850	*Lactobacillus frumenti*	NZ_AZER01000001.1	9E-144	98%
871	*Coprococcus comes*	NZ_GG662006.1	0	98%
872	[*Clostridium*] *asparagiforme*	NZ_GG657593.1	2E-170	94%
883	*Catonella morbi*	NZ_KI535368.1	1E-142	90%
890	*Massilioclostridium coli*	NZ_FMIZ01000006.1	2E-159	93%
891	*Eubacterium sp. Marseille*	NZ_LT635479.1	4E-167	94%
892	*Acetivibrio ethanolgignens*	NZ_LNAM01000152.1	5E-166	94%
893	*Mitsuokella multacida*	NZ_GG697141.2	7E-145	98%
1001	*Eubacterium xylanophilum*	NZ_JAEB01000051.1	2E-174	95%

**BLAST search was performed for the selected operational taxonomic units (OTU) (>0.01% relative abundance).*

†NCBI Reference Sequence database (https://blast.ncbi.nlm.nih.gov/Blast.cgi?PAGE_TYPE=BlastSearch&BLAST_SPEC=MicrobialGenomes); last accessed 25/1/2019.

TABLE S3 Relative abundance (%) of most abundant phyla (>0.01% of all reads) in feces of pigs fed diets with or without phytase and lactic acid treatment of cereals

	**No phytase**		**Phytase**			***p*-value**[1]		
**Treatment of cereal grains**	**Con**	**LA**	**Con**	**LA**	**SEM**	**Phytase**	**LA**	**Phytase × LA**
Firmicutes	80.40	76.67	82.60	75.52	3.837	0.893	0.172	0.666
Euryarchaeota	13.20	14.05	12.90	11.86	2.420	0.611	0.969	0.700
Bacteroidetes	3.01	3.92	2.19	7.83	1.992	0.445	0.114	0.248
Actinobacteria	1.61	2.99	0.68	2.00	0.634	0.144	0.043	0.964
Spirochaetes	0.46	1.03	0.49	1.81	0.539	0.4 51	0.093	0.495
Proteobacteria	1.02	1.03	0.93	0.64	0.197	0.229	0.490	0.470
Unassigned	0.19	0.23	0.10	0.17	0.085	0.403	0.509	0.837
Planctomycetes	0.017	0.019	0.030	0.026	0.012	0.438	0.923	0.769
Cyanobacteria	0.016	0.013	0.012	0.035	0.008	0.252	0.217	0.095
Synergistetes	0.021	0.020	0.017	0.007	0.007	0.232	0.434	0.506

Values are presented as least square means ± SEM (n = 8 pigs per dietary treatment; with exception of Con diet with phytase group, n = 7 pigs). Con, control diet; LA, diet containing lactic acid-treated cereals. [1]*p*-values for fixed effects (phytase, LA treatment of cereals and their two-way interaction) were computed using ANOVA and the PROC MIXED in SAS.

TABLE S4 Relative abundance (%) of most abundant genera (>0.05% of all reads) in feces of pigs fed diets with or without phytase and lactic acid treatment of cereals

	**No phytase**		**Phytase**			***p*-value**[1]		
**Treatment of cereal grains**	**Con**	**LA**	**Con**	**LA**	**SEM**	**Phytase**	**LA**	**Phytase × LA**
Lactobacillus	25.88	25.91	16.23	12.43	4.703	0.022	0.693	0.688
Unclassified *Clostridiaceae* 1	3.89	5.89	9.52	16.10	2.709	0.008	0.126	0.407
Unclassified *Ruminococcaceae* 1	10.98	7.90	9.61	6.19	1.272	0.238	0.017	0.897
Unclassified *Clostridiaceae* 2	4.23	9.83	6.12	14.34	2.984	0.294	0.030	0.664
Unclassified *Lachnospiraceae* 1	7.16	5.00	9.89	5.48	1.178	0.188	0.010	0.349
Ruminococcus	0.15[b]	0.10[b]	0.30[a]	0.07[b]	0.041	0.169	0.002	0.040
Unclassified *Clostridiales* 3	3.07	2.11	3.69	2.28	0.513	0.446	0.031	0.672
Clostridium	0.95	0.75	2.12	2.24	0.478	0.428	0.046	0.377
Dorea	1.88	0.86	2.11	0.87	0.304	0.011	0.933	0.739
Unclassified *Coriobacteriaceae*	1.10	2.22	0.53	1.46	0.465	0.699	0.001	0.706
Phascolarctobacterium	1.08	0.33	1.23	0.35	0.226	0.164	0.037	0.838
Unclassified *Peptostreptococcaceae*	0.72	0.20	0.76	0.36	0.116	0.692	0.002	0.777
[*Eubacterium*]	0.29	0.59	0.03	0.91	0.263	0.397	0.001	0.583
VadinCA11	0.38	0.14	0.50	0.25	0.099	0.912	0.033	0.284
Dialister	0.08	0.31	0.01	0.41	0.115	0.256	0.021	0.970
[*Ruminococcus*]	0.15	0.10	0.30	0.07	0.041	0.240	0.015	0.896
Dehalobacterium	0.040	0.066	0.070	0.037	0.012	0.007	0.940	0.217

Values are presented as least square means ± SEM (n = 8 pigs per dietary treatment; with exception of Con diet with phytase group, n = 7 pigs). Con, control diet; LA, diet containing lactic acid-treated cereals. [a,b] Different superscripts within a row indicate significant

difference ($p \leq 0.05$). [1] *p*-values for fixed effects (phytase, LA treatment of cereals and their two-way interaction) were computed using ANOVA and the PROC MIXED in SAS.

TABLE S5 Relative abundance of selected operational taxonomic units (OTUs) detected in feces of pigs fed diets with or without phytase and lactic acid treatment of cereals

**OTU (sequence reads)**[1]	**Sequence Taxonomy (genus/species)**	**No phytase**		**Phytase**			***p*-value**[2]		
		**Con**	**LA**	**Con**	**LA**	**SEM**	**Phytase**	**LA**	**Phytase × LA**
OTU4 (3398.00)	*Lactobacillus*	7.04	10.97	3.69	5.50	2.046	0.042	0.174	0.609
OTU6 (1373.44)	*Methanobrevibacter*	4.69	3.22	2.04	2.52	1.552	0.292	0.752	0.537
OTU12 (561.75)	*Ruminococcus flavefaciens*	2.09	1.28	1.21	0.72	0.515	0.175	0.220	0.755
OTU 14 (494.75)	*Lactobacillus*	1.49	0.39	2.39	0.34	0.499	0.406	0.004	0.352
OTU18 (390.72)	*Clostridium*	0.43	0.24	0.86	1.15	0.255	0.014	0.837	0.353
OTU19 (375.75)	Unclassified *Ruminococcaceae*	0.72[b]	0.41[b]	2.00[a]	0.30[b]	0.249	0.027	0.001	0.010
OTU21 (301.50)	Unclassified *Ruminococcaceae*	1.30	0.50	0.54	0.33	0.195	0.025	0.016	0.144
OTU23 (283.16)	*Dorea*	0.74	0.41	0.80	0.44	0.145	0.756	0.029	0.912
OTU25 (263.06)	*Prevotella*	0.23	0.48	0.06	1.65	0.462	0.292	0.058	0.161
OTU27 (261.09)	Unclassified *Lachnospiraceae*	0.71	0.19	1.06	0.26	0.214	0.342	0.005	0.535
OTU34 (214.06)	Unclassified *Ruminococcaceae*	0.48	0.85	0.13	0.26	0.171	0.011	0.150	0.484
OTU35 (209.84)	Unclassified *Peptostreptococcaceae*	0.69	0.17	0.69	0.27	0.117	0.671	<0.001	0.673
OTU36 (192.75)	[*Eubacterium*] *cylindroides*	0.23	0.54	0.01	0.84	0.247	0.876	0.031	0.296
OTU38 (186.65)	*Clostridium*	0.22	0.03	0.78	0.19	0.264	0.183	0.155	0.453

OTU39 (184.66)	*Lactobacillus*	0.32	0.13	1.17	0.17	0.225	0.061	0.014	0.084
OTU41 (173.53)	*Collinsella aerofaciens*	0.22	0.52	0.07	0.47	0.165	0.552	0.048	0.769
OTU44 (170.41)	*Prevotella copri*	0.17	0.26	0.21	1.01	0.282	0.174	0.127	0.221
OTU49 (150.56)	*Phascolarctobacterium*	0.56	0.03	0.54	0.10	0.106	0.829	<0.001	0.668
OTU55 (105.29)	*Prevotella*	0.14	0.30	0.03	0.57	0.169	0.636	0.049	0.263
OTU58 (108.31)	Unclassified *Clostridiaceae*	0.11	0.09	0.20	0.38	0.082	0.031	0.332	0.254
OTU60 (105.59)	*Dorea*	0.30	0.16	0.34	0.13	0.058	0.897	0.006	0.610
OTU66 (95.78)	Unclassified *Lachnospiraceae*	0.17	0.13	0.45	0.11	0.084	0.127	0.033	0.094
OTU69 (92.31)	*VadinCA11*	0.13	0.03	0.34	0.15	0.089	0.080	0.131	0.610
OTU70 (91.53)	*Dialister*	0.08	0.30	0.01	0.34	0.098	0.894	0.010	0.623
OTU71 (88.96)	Unclassified *Clostridiales*	0.18	0.08	0.22	0.22	0.048	0.073	0.353	0.285
OTU82 (75.65)	*Ruminococcus*	0.43	0.05	0.11	0.08	0.110	0.198	0.072	0.125
OTU85 (75.00)	*Peptococcus*	0.14	0.30	0.08	0.09	0.055	0.019	0.154	0.200
OTU92 (71.00)	Unclassified *Ruminococcaceae*	0.15	0.13	0.11	0.24	0.053	0.530	0.281	0.155
OTU93 (70.63)	*Dorea formicigenerans*	0.22	0.07	0.19	0.09	0.049	0.925	0.021	0.625
OTU94 (69.53)	Unclassified *Lachnospiraceae*	0.20	0.08	0.30	0.05	0.051	0.539	0.001	0.211
OTU95 (69.34)	*Coprococcus*	0.10	0.01	0.37	0.08	0.066	0.017	0.008	0.140

OTU98 (66.84)	Unclassified *Lachnospiraceae*	0.14	0.02	0.27	0.13	0.068	0.089	0.068	0.885
OTU99 (66.28)	*Oscillospira*	0.16	0.02	0.27	0.07	0.077	0.292	0.038	0.670
OTU102 (64.97)	Unclassified *Clostridiales*	0.29	0.05	0.28	0.02	0.102	0.809	0.022	0.899
OTU114 (61.34)	Unclassified *Christensenellaceae*	0.20	0.11	0.15	0.05	0.047	0.293	0.057	0.885
OTU123 (55.53)	Unclassified *Lachnospiraceae*	0.023	0.234	0.000	0.122	0.074	0.372	0.034	0.564
OTU124 (55.46)	Unclassified *Ruminococcaceae*	0.168	0.140	0.099	0.049	0.045	0.087	0.394	0.805
OTU125 (55.34)	Unclassified *Clostridiales*	0.019	0.209	0.007	0.204	0.078	0.914	0.020	0.964
OTU131 (50.13)	*VadinCA11*	0.172	0.088	0.098	0.075	0.023	0.066	0.027	0.195
OTU133 (47.91)	Unclassified *Christensenellaceae*	0.154	0.154	0.087	0.036	0.038	0.022	0.497	0.504
OTU134 (47.31)	Unclassified *Ruminococcaceae*	0.147	0.004	0.259	0.036	0.071	0.324	0.017	0.577
OTU139 (45.40)	Unclassified *Lachnospiraceae*	0.111	0.003	0.171	0.069	0.039	0.115	0.013	0.932
OTU143 (42.16)	Unclassified *Coriobacteriaceae*	0.099	0.088	0.075	0.076	0.019	0.344	0.810	0.766
OTU144 (41.84)	*Blautia*	0.085	0.019	0.212	0.053	0.027	0.007	<0.001	0.099
OTU147 (41.22)	Unclassified *Lachnospiraceae*	0.275	0.002	0.067	0.035	0.092	0.355	0.112	0.206
OTU150 (40.94)	Unclassified *Coriobacteriaceae*	0.108[a]	0.073[ab]	0.022[b]	0.113[a]	0.027	0.388	0.295	0.026
OTU155 (39.47)	Unclassified *Clostridiaceae*	0.025	0.042	0.069	0.131	0.028	0.025	0.165	0.424
OTU157 (38.62)	*Coprococcus eutactus*	0.185	0.066	0.031	0.049	0.050	0.100	0.323	0.183

OTU161 (34.84)	Unclassified *Clostridiaceae*	0.012	0.036	0.066	0.121	0.025	0.012	0.134	0.562
OTU162 (34.53)	Unclassified [*Mogibacteriaceae*]	0.067	0.034	0.102	0.070	0.017	0.056	0.078	0.971
OTU168 (33.25)	*Coprococcus*	0.017	0.006	0.127	0.061	0.040	0.051	0.347	0.500
OTU174 (31.81)	Unclassified *Clostridiaceae*	0.036	0.017	0.065	0.096	0.022	0.021	0.785	0.250
OTU175 (31.56)	Unclassified *Ruminococcaceae*	0.133	0.004	0.097	0.014	0.050	0.790	0.043	0.643
OTU176 (31.16)	Unclassified *Veillonellaceae*	0.041	0.137	0.015	0.076	0.034	0.203	0.028	0.605
OTU178 (30.90)	[*Ruminococcus*]	0.096	0.046	0.109	0.029	0.030	0.949	0.042	0.625
OTU183 (30.37)	Unclassified *Coriobacteriaceae*	0.050	0.080	0.004	0.114	0.062	0.917	0.269	0.520
OTU192 (28.53)	*Lactobacillus*	0.059	0.136	0.093	0.002	0.075	0.512	0.928	0.281
OTU193 (28.31)	Unclassified *Clostridiales*	0.035	0.042	0.041	0.085	0.018	0.201	0.176	0.330
OTU197 (27.91)	*Blautia*	0.020	0.041	0.027	0.131	0.052	0.353	0.235	0.432
OTU205 (26.78)	*Methanosphaera*	0.001	0.145	0.000	0.117	0.098	0.842	0.181	0.933
OTU207 (26.37)	*Blautia*	0.050	0.054	0.063	0.044	0.025	0.949	0.760	0.641
OTU222 (19.12)	*Megasphaera*	0.014	0.093	0.013	0.066	0.030	0.646	0.037	0.670
OTU225 (18.28)	*Prevotella*	0.039	0.054	0.037	0.047	0.025	0.858	0.632	0.921
OTU227 (18.03)	Unclassified *Ruminococcaceae*	0.065	0.042	0.021	0.023	0.009	0.002	0.263	0.177
OTU228 (18.00)	Unclassified *Lachnospiraceae*	0.058	0.029	0.064	0.011	0.011	0.579	0.001	0.275

OTU230 (17.91)	[*Prevotella*]	0.036	0.035	0.033	0.084	0.034	0.501	0.475	0.459
OTU236 (17.47)	*Lactobacillus*	0.041	0.013	0.099	0.013	0.017	0.106	0.003	0.102
OTU238 (17.12)	Unclassified *Ruminococcaceae*	0.035	0.050	0.049	0.013	0.016	0.485	0.520	0.135
OTU240 (16.97)	Unclassified *Lachnospiraceae*	0.059	0.023	0.030	0.023	0.007	0.064	0.009	0.059
OTU241 (16.93)	Unclassified *Ruminococcaceae*	0.012	0.136	0.030	0.026	0.070	0.515	0.404	0.370
OTU248 (16.62)	Unclassified *Lachnospiraceae*	0.035	0.009	0.045	0.041	0.016	0.205	0.358	0.494
OTU254 (15.93)	Unclassified [*Mogibacteriaceae*]	0.050	0.050	0.013	0.001	0.019	0.038	0.766	0.754
OTU260 (15.16)	Unclassified *Lachnospiraceae*	0.025	0.009	0.054	0.026	0.010	0.033	0.041	0.527
OTU264 (14.81)	*Dorea*	0.035	0.019	0.046	0.025	0.008	0.321	0.025	0.769
OTU267 (14.72)	Unclassified [*Mogibacteriaceae*]	0.036	0.022	0.052	0.010	0.013	0.875	0.039	0.289
OTU269 (14.38)	Unclassified *Ruminococcaceae*	0.050	0.039	0.013	0.015	0.014	0.043	0.767	0.657
OTU270 (14.16)	Unclassified *Lachnospiraceae*	0.044	0.013	0.068	0.008	0.014	0.503	0.003	0.302
OTU271 (14.09)	Unclassified *Ruminococcaceae*	0.018	0.030	0.022	0.036	0.006	0.446	0.044	0.889
OTU280 (13.25)	*Prevotella*	0.012	0.034	0.000	0.068	0.022	0.606	0.048	0.303
OTU281 (13.19)	*Prevotella*	0.040[ab]	0.012[b]	0.006[b]	0.072[a]	0.019	0.477	0.328	0.019
OTU284 (13.16)	*Coprococcus*	0.040	0.014	0.054	0.010	0.012	0.688	0.008	0.482
OTU293 (12.53)	*Blautia*	0.008	0.000	0.070	0.021	0.019	0.041	0.155	0.292

OTU298 (12.28)	Unclassified *Lachnospiraceae*	0.019	0.016	0.069	0.012	0.013	0.090	0.032	0.053
OTU301 (12.00)	Unclassified *Clostridiales*	0.042	0.009	0.073	0.000	0.022	0.613	0.024	0.388
OTU303 (11.97)	Unclassified [*Mogibacteriaceae*]	0.005	0.048	0.001	0.051	0.018	0.979	0.019	0.859
OTU305 (11.81)	*VadinCA11*	0.013	0.006	0.068	0.009	0.015	0.064	0.041	0.095
OTU306 (11.81)	*VadinCA11*	0.070[a]	0.013[b]	0.000[b]	0.018[ab]	0.018	0.074	0.291	0.044
OTU307 (11.71)	Unclassified *Clostridiales*	0.010	0.049	0.031	0.007	0.015	0.507	0.610	0.046
OTU315 (11.43)	*Campylobacter*	0.052	0.042	0.004	0.002	0.017	0.018	0.741	0.822
OTU318 (11.38)	Unclassified *Ruminococcaceae*	0.024	0.013	0.051	0.013	0.011	0.226	0.039	0.244
OTU319 (11.28)	Unclassified *Clostridiales*	0.030	0.012	0.052	0.011	0.012	0.376	0.018	0.356
OTU329 (10.71)	*Oscillospira*	0.015	0.043	0.009	0.020	0.009	0.116	0.047	0.369
OTU331 (10.66)	*Blautia*	0.042	0.026	0.008	0.016	0.010	0.038	0.699	0.241
OTU348 (10.06)	Unclassified *Lachnospiraceae*	0.031	0.015	0.035	0.009	0.007	0.894	0.006	0.509
OTU362 (9.63)	Unclassified [*Mogibacteriaceae*]	0.032	0.009	0.044	0.006	0.703	0.014	0.525	0.703
OTU368 (9.47)	*Bulleidia p-1630-c5*	0.007	0.020	0.002	0.046	0.338	0.016	0.174	0.338
OTU370 (9.38)	Unclassified *Ruminococcaceae*	0.003	0.025	0.038	0.005	0.507	0.600	0.023	0.507
OTU371 (9.37)	*rc4-4*	0.010	0.003	0.028	0.019	0.008	0.049	0.323	0.879
OTU375 (9.16)	Unclassified *Clostridiaceae*	0.008	0.010	0.016	0.029	0.006	0.041	0.239	0.397

OTU377 (9.13)	Unclassified *Lachnospiraceae*	0.023	0.011	0.042	0.014	0.006	0.097	0.005	0.228
OTU380 (9.06)	Unclassified *Clostridiales*	0.042	0.011	0.028	0.005	0.010	0.321	0.010	0.712
OTU382 (8.97)	Unclassified *Ruminococcaceae*	0.024	0.037	0.005	0.009	0.009	0.012	0.352	0.582
OTU387 (8.81)	*Dehalobacterium*	0.010[ab]	0.022[ab]	0.031[a]	0.003[b]	0.009	0.910	0.361	0.040
OTU389 (8.72)	Unclassified *Clostridiales*	0.019	0.005	0.060	0.004	0.016	0.219	0.035	0.188
OTU395 (8.59)	Unclassified [*Mogibacteriaceae*]	0.016	0.011	0.034	0.008	0.007	0.276	0.029	0.125
OTU400 (8.47)	Unclassified *Clostridiaceae*	0.009	0.007	0.016	0.029	0.006	0.027	0.401	0.221
OTU413 (8.06)	Unclassified *Clostridiales*	0.036	0.013	0.017	0.004	0.007	0.063	0.015	0.497
OTU416 (7.94)	Unclassified *Clostridiales*	0.005	0.007	0.021	0.029	0.009	0.035	0.527	0.740
OTU417 (7.91)	*Oscillospira*	0.040	0.005	0.015	0.008	0.009	0.221	0.028	0.134
OTU419 (7.88)	Unclassified *Coriobacteriaceae*	0.024	0.028	0.002	0.010	0.003	<0.001	0.084	0.609
OTU420 (7.84)	*Clostridium*	0.005	0.005	0.014	0.028	0.007	0.042	0.349	0.372
OTU423 (7.78)	Unclassified *Lachnospiraceae*	0.019	0.005	0.031	0.008	0.008	0.311	0.024	0.576
OTU442 (7.38)	*Dorea*	0.017	0.009	0.032	0.008	0.007	0.270	0.025	0.241
OTU443 (7.34)	*Coprococcus*	0.021	0.002	0.036	0.012	0.010	0.206	0.036	0.766
OTU444 (7.34)	*Blautia*	0.020	0.011	0.022	0.010	0.005	0.916	0.041	0.720
OTU446 (7.19)	*Slackia*	0.010	0.020	0.005	0.018	0.005	0.458	0.029	0.692

OTU449 (7.06)	Unclassified *Lachnospiraceae*	0.016	0.001	0.030	0.011	0.007	0.108	0.030	0.771
OTU458 (6.81)	Unclassified *Desulfovibrionaceae*	0.025	0.016	0.014	0.008	0.004	0.033	0.075	0.706
OTU460 (6.78)	Unclassified *Clostridiaceae*	0.005	0.007	0.012	0.024	0.004	0.007	0.085	0.235
OTU462 (6.78)	*Lactobacillus*	0.018	0.006	0.030	0.003	0.007	0.521	0.009	0.273
OTU467 (6.66)	Unclassified *Ruminococcaceae*	0.011	0.007	0.034	0.010	0.005	0.019	0.010	0.051
OTU474 (6.56)	*Oscillospira*	0.023	0.001	0.023	0.006	0.008	0.744	0.024	0.716
OTU484 (6.28)	Unclassified *Lachnospiraceae*	0.022	0.011	0.016	0.008	0.004	0.311	0.025	0.740
OTU485 (6.25)	Unclassified *Lachnospiraceae*	0.013	0.002	0.031	0.003	0.008	0.245	0.021	0.271
OTU489 (6.19)	Unclassified *Ruminococcaceae*	0.018	0.006	0.019	0.009	0.004	0.587	0.017	0.839
OTU492 (6.13)	Unclassified *Veillonellaceae*	0.010	0.016	0.004	0.022	0.006	0.979	0.045	0.298
OTU495 (6.06)	*Dorea*	0.020	0.006	0.019	0.010	0.004	0.659	0.004	0.570
OTU510 (5.84)	Unclassified *Lachnospiraceae*	0.018	0.004	0.027	0.004	0.005	0.397	0.002	0.425
OTU513 (5.78)	Unclassified *Coriobacteriaceae*	0.007	0.012	0.000	0.024	0.006	0.725	0.029	0.158
OTU516 (5.75)	Unclassified *Lachnospiraceae*	0.011	0.001	0.025	0.010	0.005	0.032	0.023	0.659
OTU517 (5.72)	Unclassified *Ruminococcaceae*	0.014	0.000	0.033	0.005	0.008	0.164	0.015	0.370
OTU519 (5.69)	Unclassified *Clostridiales*	0.005	0.001	0.017	0.016	0.005	0.017	0.618	0.719
OTU524 (5.63)	*Oscillospira*	0.019	0.001	0.021	0.006	0.007	0.621	0.031	0.901

OTU535 (5.41)	Unclassified *Lachnospiraceae*	0.019	0.002	0.024	0.003	0.005	0.606	0.002	0.656
OTU536 (5.38)	Unclassified *Clostridiaceae*	0.002	0.006	0.013	0.019	0.005	0.038	0.326	0.904
OTU540 (5.22)	Unclassified *Ruminococcaceae*	0.017	0.002	0.021	0.004	0.007	0.670	0.034	0.907
OTU547 (5.13)	Unclassified *Ruminococcaceae*	0.007	0.016	0.003	0.022	0.006	0.866	0.033	0.401
OTU553 (5.03)	Unclassified *Clostridiales*	0.008	0.002	0.023	0.008	0.003	0.006	0.005	0.224
OTU557 (4.97)	*Lactobacillus*	0.012	0.016	0.006	0.006	0.003	0.040	0.600	0.608
OTU568 (4.75)	Unclassified *Clostridiaceae*	0.004	0.004	0.008	0.018	0.004	0.021	0.156	0.199
OTU575 (4.72)	Unclassified *Clostridiaceae*	0.002	0.006	0.010	0.017	0.004	0.018	0.148	0.561
OTU580 (4.66)	Unclassified *Lachnospiraceae*	0.016	0.005	0.017	0.006	0.004	0.807	0.010	0.991
OTU581 (4.66)	Unclassified *Veillonellaceae*	0.006	0.022	0.002	0.013	0.004	0.119	0.005	0.570
OTU588 (4.56)	Unclassified *Lachnospiraceae*	0.016	0.001	0.012	0.008	0.004	0.797	0.045	0.196
OTU590 (4.56)	Unclassified *Lachnospiraceae*	0.015	0.006	0.020	0.004	0.006	0.851	0.034	0.550
OTU594 (4.53)	Unclassified *Clostridiales*	0.017	0.002	0.025	0.001	0.008	0.673	0.024	0.555
OTU600 (4.41)	Unclassified *Ruminococcaceae*	0.006	0.004	0.018	0.007	0.004	0.041	0.093	0.211
OTU608 (4.34)	*Lactobacillus*	0.008	0.002	0.029	0.002	0.006	0.074	0.009	0.069
OTU610 (4.28)	Unclassified *Clostridiaceae*	0.002	0.004	0.008	0.014	0.004	0.033	0.265	0.586
OTU619 (4.19)	*Dorea*	0.015	0.008	0.015	0.003	0.004	0.535	0.032	0.606

OTU620 (4.19)	*Ruminococcus*	0.024	0.003	0.010	0.002	0.006	0.250	0.029	0.327
OTU623 (4.13)	Unclassified *Lachnospiraceae*	0.009	0.002	0.019	0.004	0.004	0.099	0.007	0.307
OTU630 (4.06)	*Lactobacillus*	0.004	0.001	0.039	0.001	0.009	0.071	0.038	0.073
OTU640 (3.97)	Unclassified *Clostridiales*	0.018	0.001	0.021	0.001	0.006	0.779	0.005	0.759
OTU660 (3.81)	Unclassified *Lachnospiraceae*	0.009	0.002	0.017	0.005	0.004	0.183	0.026	0.602
OTU661 (3.81)	Unclassified *Ruminococcaceae*	0.005	0.003	0.019	0.003	0.004	0.124	0.044	0.130
OTU662 (3.81)	*Oscillospira*	0.005	0.014	0.006	0.007	0.002	0.172	0.028	0.055
OTU680 (3.59)	Unclassified *Clostridiales*	0.010	0.005	0.015	0.002	0.003	0.649	0.016	0.233
OTU690 (3.53)	Unclassified *Ruminococcaceae*	0.008	0.002	0.018	0.003	0.004	0.209	0.016	0.235
OTU691 (3.5)	*Peptococcus*	0.005^{b}	0.015^{ab}	0.008^{ab}	0.003^{b}	0.003	0.200	0.420	0.041
OTU704 (3.38)	Unclassified *Clostridiales*	0.003	0.009	0.002	0.014	0.004	0.464	0.015	0.426
OTU720 (3.25)	Unclassified *Clostridiaceae*	0.002	0.004	0.005	0.011	0.011	0.044	0.064	0.377
OTU728 (3.19)	Unclassified *Clostridiales*	0.019	0.002	0.009	0.002	0.005	0.270	0.019	0.311
OTU729 (3.19)	Unclassified *Ruminococcaceae*	0.001	0.001	0.002	0.017	0.004	0.037	0.079	0.062
OTU735 (3.16)	*Blautia*	0.012	0.001	0.015	0.003	0.004	0.475	0.009	0.930
OTU741 (3.13)	Unclassified *Clostridiales*	0.004	0.005	0.002	0.011	0.002	0.342	0.036	0.158
OTU747 (3.09)	Unclassified *Clostridiales*	0.012	0.004	0.010	0.002	0.004	0.628	0.027	0.997

OTU748 (3.09)	*Phascolarctobacterium*	0.011	0.000	0.011	0.002	0.002	0.650	<0.001	0.742
OTU761 (3.03)	Unclassified *Ruminococcaceae*	0.005	0.009	0.002	0.008	0.002	0.394	0.037	0.806
OTU779 (2.94)	Unclassified *Lachnospiraceae*	0.010	0.002	0.015	0.001	0.003	0.489	0.004	0.379
OTU789 (2.91)	Unclassified *Veillonellaceae*	0.003	0.010	0.002	0.010	0.002	0.863	0.004	0.863
OTU797 (2.88)	Unclassified *Lachnospiraceae*	0.005	0.004	0.013	0.002	0.002	0.174	0.012	0.058
OTU820 (2.78)	Unclassified *Lachnospiraceae*	0.002	0.012	0.003	0.006	0.003	0.383	0.044	0.245
OTU822 (2.75)	Unclassified *Lachnospiraceae*	0.007	0.001	0.015	0.003	0.003	0.119	0.010	0.415
OTU829 (2.72)	Unclassified *Ruminococcaceae*	0.009	0.003	0.012	0.003	0.002	0.641	<0.001	0.422
OTU835 (2.69)	Unclassified *Lachnospiraceae*	0.009	0.000	0.013	0.002	0.003	0.273	0.001	0.819
OTU837 (2.69)	Unclassified *Clostridiales*	0.009	0.002	0.011	0.003	0.003	0.595	0.012	0.683
OTU848 (2.63)	Unclassified *Lachnospiraceae*	0.005	0.002	0.012	0.004	0.001	0.004	0.001	0.061
OTU850 (2.63)	*Lactobacillus*	0.007	0.001	0.014	0.003	0.003	0.109	0.007	0.410
OTU871 (2.50)	*Dorea*	0.008	0.004	0.010	0.001	0.002	0.931	0.010	0.281
OTU872 (2.5)	Unclassified *Lachnospiraceae*	0.009	0.001	0.010	0.002	0.003	0.738	0.023	0.995
OTU883 (2.47)	Unclassified *Clostridiales*	0.004	0.001	0.016	0.002	0.003	0.052	0.015	0.080
OTU890 (2.47)	Unclassified *Ruminococcaceae*	0.007	0.004	0.008	0.003	0.001	0.912	0.005	0.316
OTU891 (2.47)	Unclassified *Lachnospiraceae*	0.007	0.005	0.007	0.002	0.002	0.458	0.045	0.557

OTU892 (2.47)	Unclassified *Lachnospiraceae*	0.002	0.009	0.002	0.009	0.003	0.924	0.025	0.959
OTU893 (2.44)	Unclassified *Veillonellaceae*	0.003	0.010	0.001	0.008	0.002	0.323	0.002	0.916
OTU1001 (2.09)	Unclassified *Clostridiales*	0.010	0.001	0.010	0.001	0.003	0.946	0.004	0.983

Values are presented as least square means ± SEM (n = 8 pigs per dietary treatment; with exception Con-Phy treatment, n = 7 pigs). [a,b] Different superscripts within a row indicate significant difference ($p \leq 0.05$). [1] Average number of sequences across all samples. Con, control diet; LA, diet containing lactic acid-treated cereals; Con-Phy, diet with phytase supplementation; and LA-Phy, diet with phytase supplementation and lactic acid-treated cereals. [2]*p*-values for fixed effects (phytase, LA treatment of cereals and their two-way interaction) were computed using ANOVA and the PROC MIXED in SAS.

TABLE S6 Permutational multivariate analysis of variance (PERMANOVA) results for fecal communities of pigs fed diets with or without phytase and lactic acid treatment of cereals

**Source of Variation**	**df**	**SS**	**R^2**	**F**	***p*-value**
Phytase	1	0.40	0.058	1.793	0.067
Lactic acid	1	0.45	0.065	2.012	0.031
Phytase : Lactic acid	1	0.08	0.012	0.363	0.990
Residual	27	6.02	0.866		
Total	30	6.95	1.000		

The analysis based on pairwise distance of a multivariate data set and values were obtained using type III sums of squares with 999 permutations of residuals, considering significant difference at $p \leq 0.05$. df, degrees of freedom; SS, sum of squares.
